# Supplementary material for: reComBat: batch-effect removal in large-scale multi-source gene-expression data integration
Source: Bioinform Adv. 2022 Oct 6;2(1):vbac071. doi: 10.1093/bioadv/vbac071 (PMC9710604; doi:10.1093/bioadv/vbac071)
Supplement: vbac071_Supplementary_Data [file vbac071_supplementary_data.zip › vbac071_supplement/supplFigures/harmDataeval.pdf]

A) Impact of Hypoxia

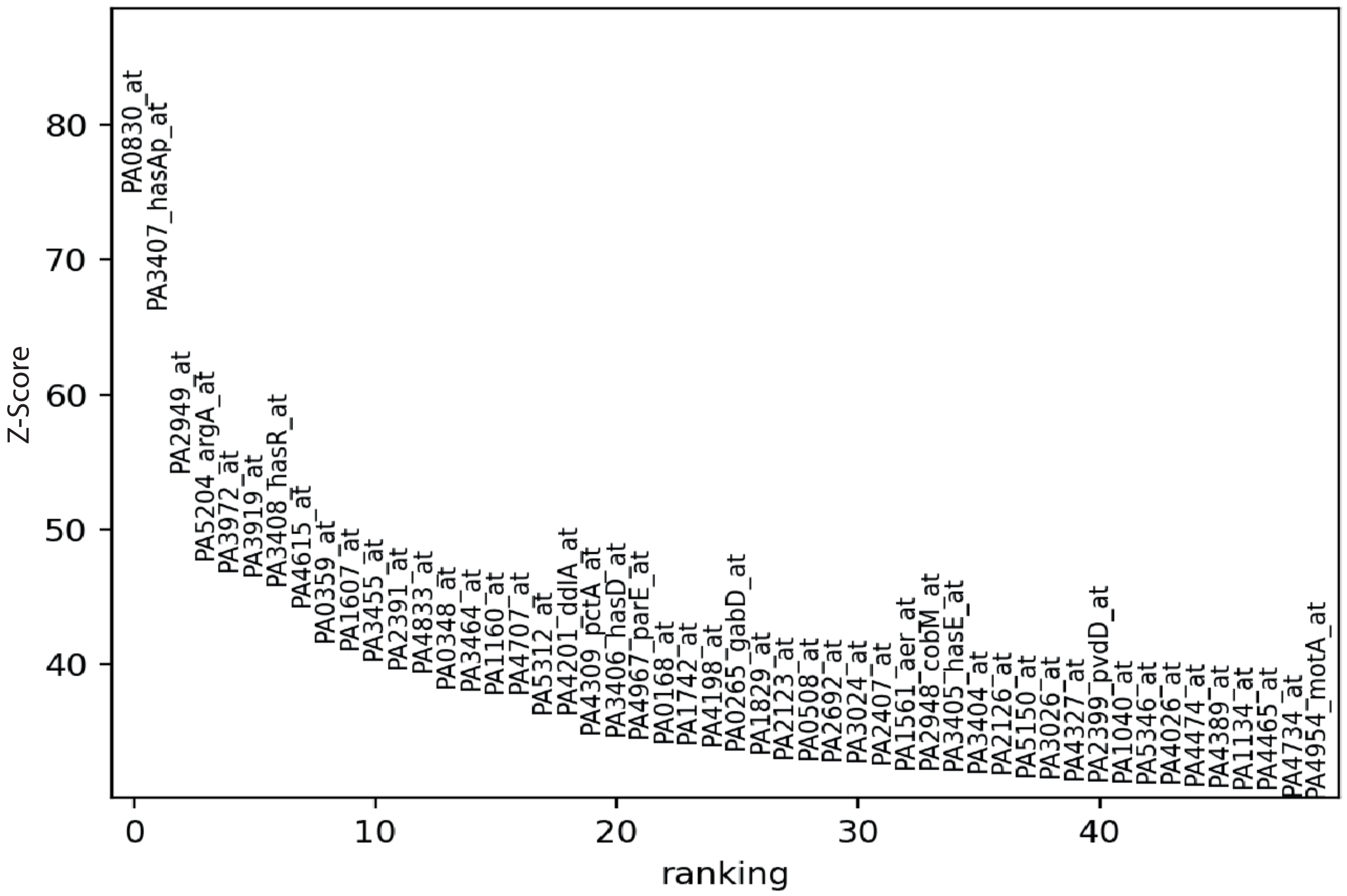

Zero-Hop	Strain	Phase	Culture	T [°C]	Oxygenation	Medium	Antibiotic	$n_{Samples}$	n_{GSE}
0	PAO1	Exp.	liquid	35-38	**hypoxic**	rich	no	48	2
35	PAO1	Exp.	liquid	35-38	**aerobic**	rich	no	170	23

B) Impact of Growth Phase

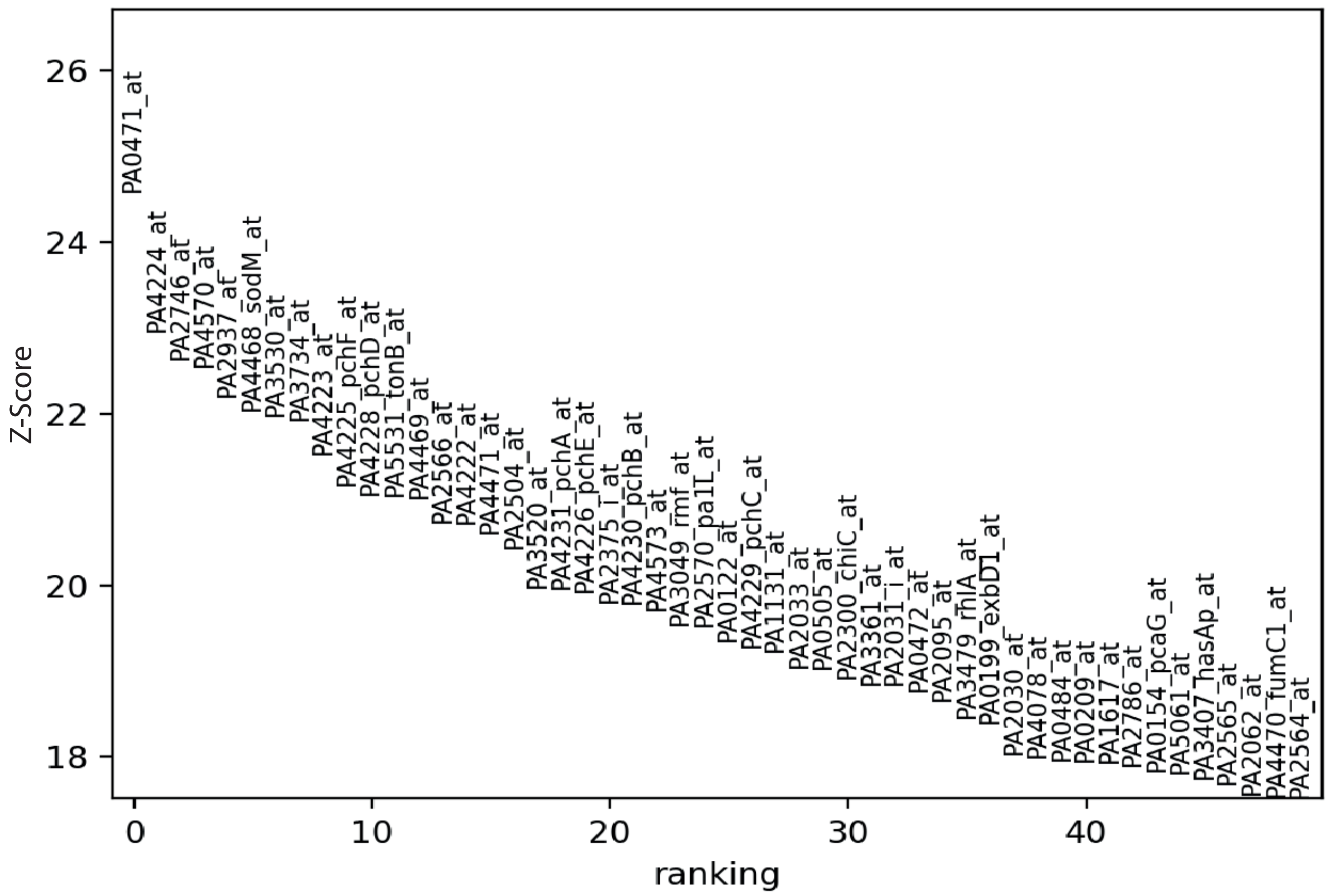

Zero-Hop	Strain	Phase	Culture	T [°C]	Oxygenation	Medium	Antibiotic	$n_{Samples}$	n_{GSE}
33	PAO1	**Plateau**	liquid	35-38	aerobic	rich	no	38	9
35	PAO1	**Exp.**	liquid	35-38	aerobic	rich	no	170	23

C) Impact of Medium richness

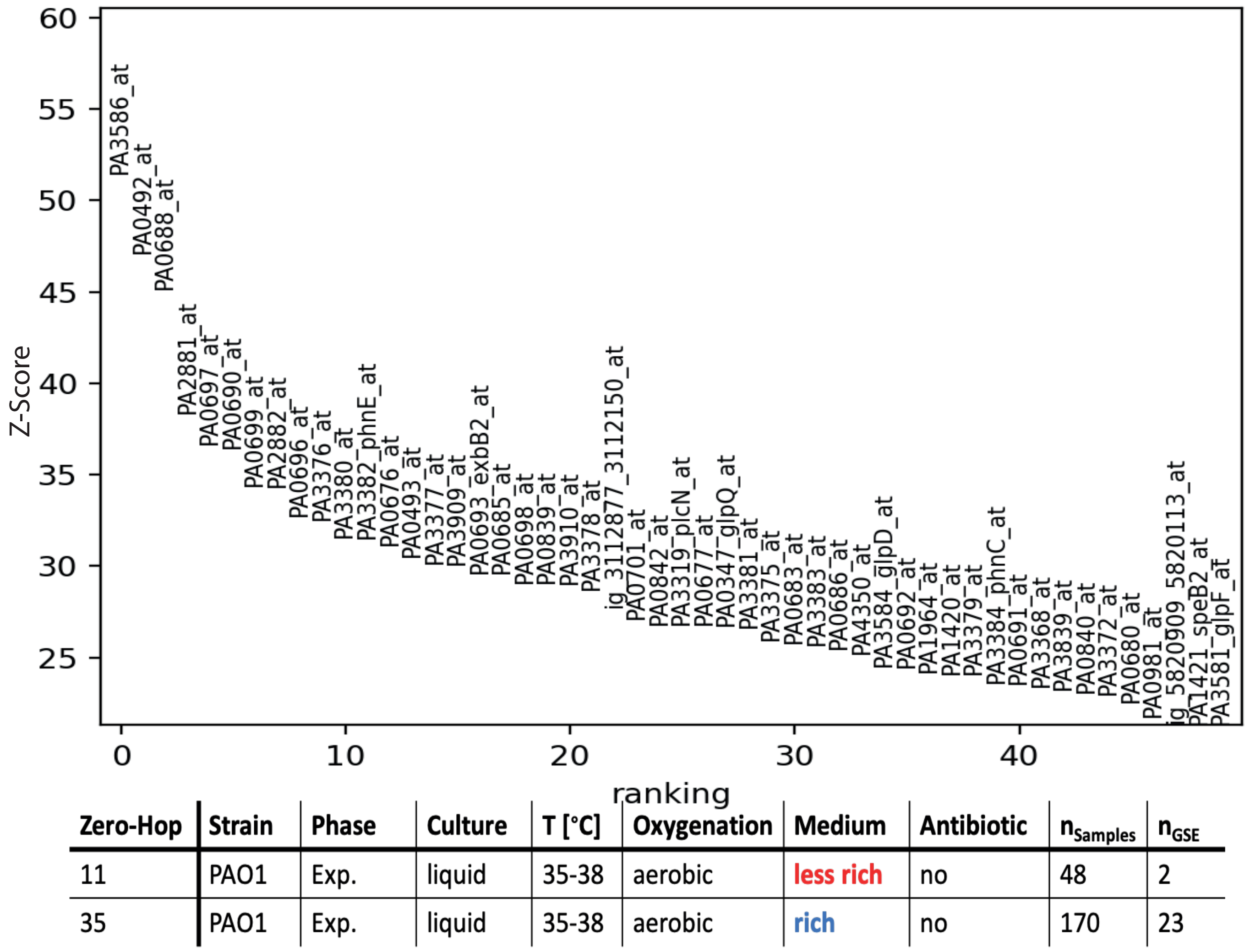

Zero-Hop	Strain	Phase	Culture	T [°C]	Oxygenation	Medium	Antibiotic	$n_{Samples}$	n_{GSE}
11	PAO1	Exp.	liquid	35-38	aerobic	**less rich**	no	48	2
35	PAO1	Exp.	liquid	35-38	aerobic	**rich**	no	170	23

D) Comparison of clinical isolates with PAO1

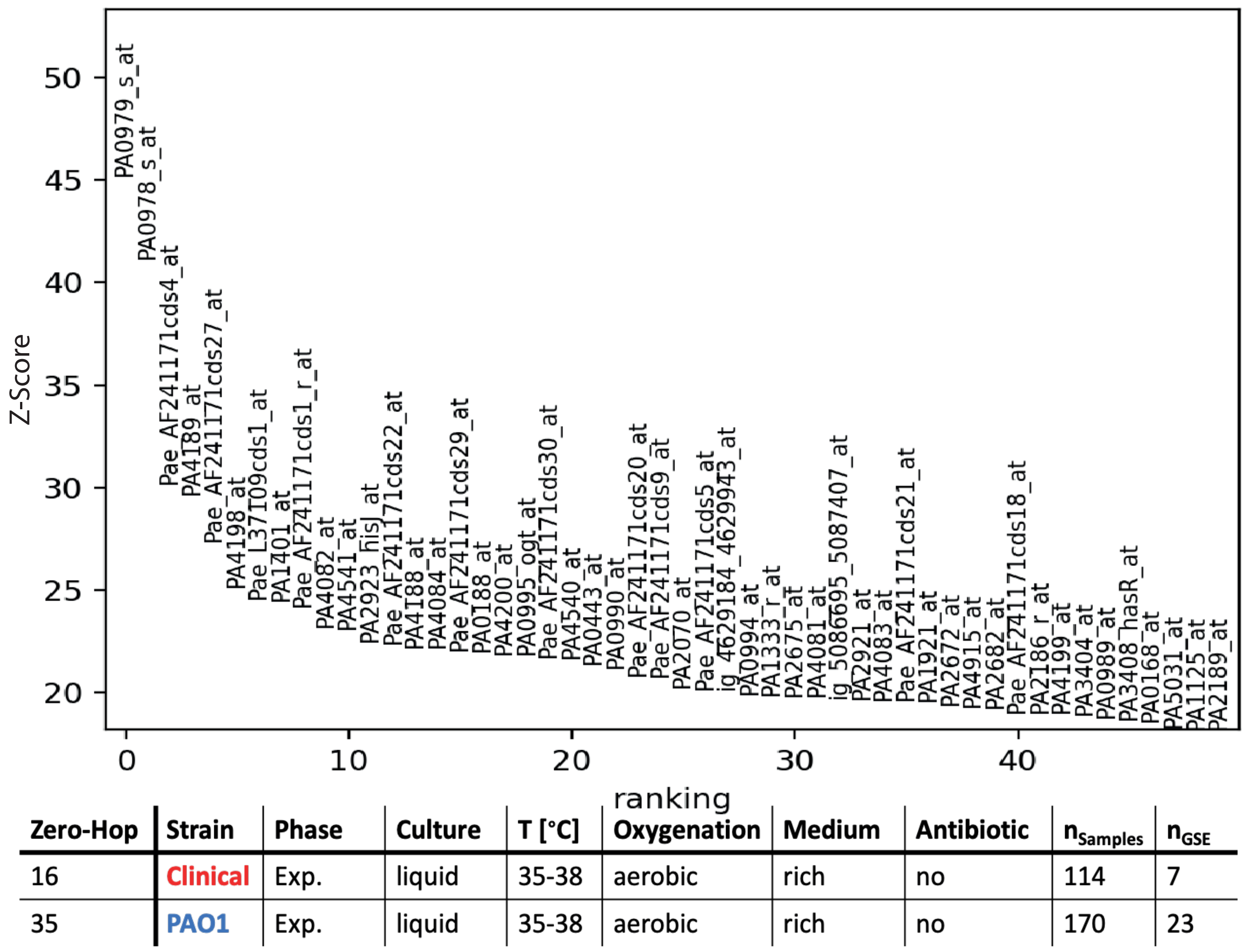

Zero-Hop	Strain	Phase	Culture	T [°C]	Oxygenation	Medium	Antibiotic	$n_{Samples}$	n_{GSE}
16	**Clinical**	Exp.	liquid	35-38	aerobic	rich	no	114	7
35	**PAO1**	Exp.	liquid	35-38	aerobic	rich	no	170	23
