# Supplementary material for: reComBat: batch-effect removal in large-scale multi-source gene-expression data integration
Source: Bioinform Adv. 2022 Oct 6;2(1):vbac071. doi: 10.1093/bioadv/vbac071 (PMC9710604; doi:10.1093/bioadv/vbac071)
Supplement: vbac071_Supplementary_Data [file vbac071_supplementary_data.zip › vbac071_supplement/supplFigures/PCmg_RNA.pdf]

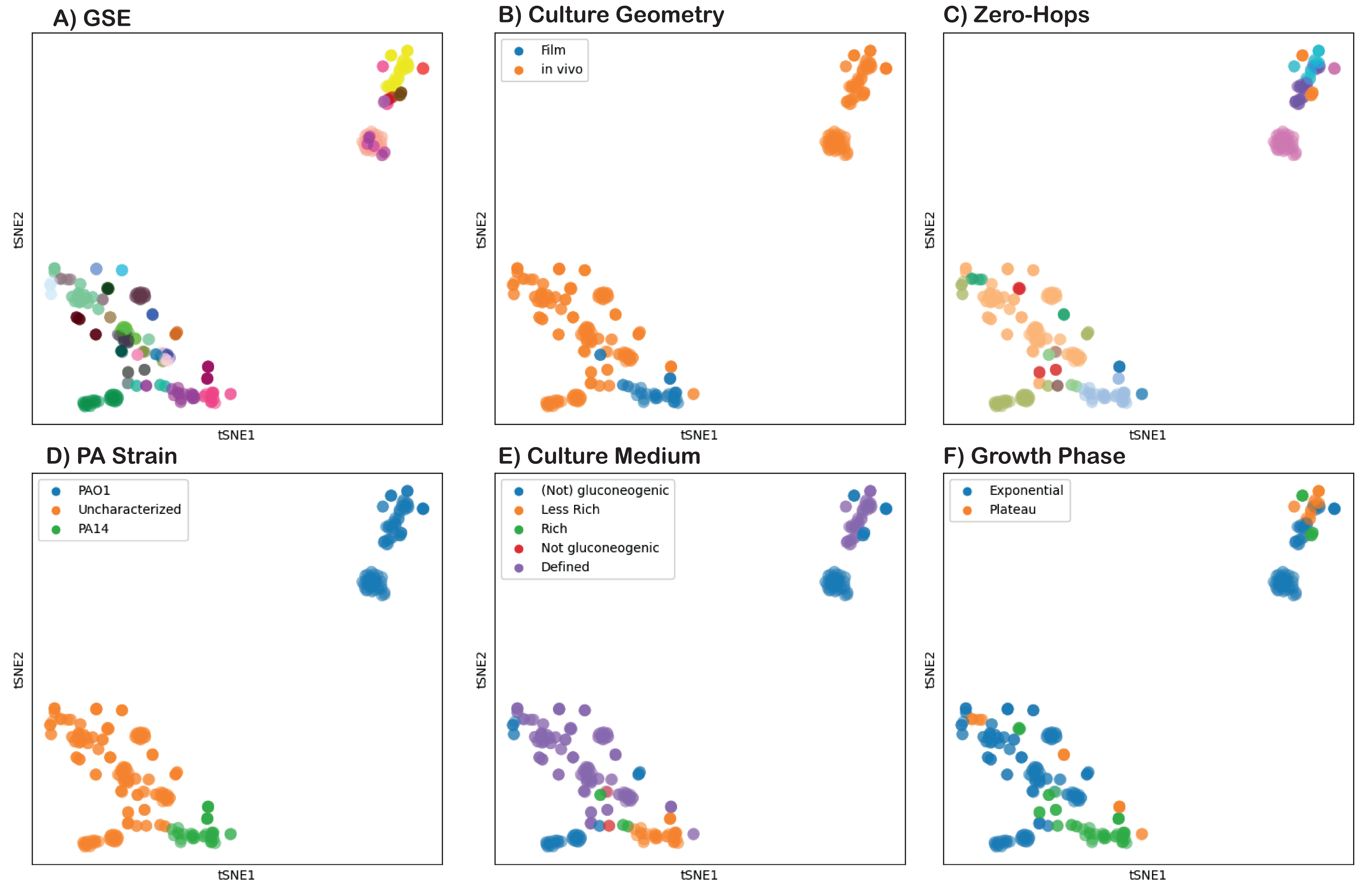
A) GSE
tSNE2
tSNE1
B) Culture Geometry
Film
in vivo
tSNE2
tSNE1
C) Zero-Hops
tSNE2
tSNE1
D) PA Strain
PAO1
Uncharacterized
PA14
tSNE2
tSNE1
E) Culture Medium
(Not) gluconeogenic
Less Rich
Rich
Not gluconeogenic
Defined
tSNE2
tSNE1
F) Growth Phase
Exponential
Plateau
tSNE2
tSNE1

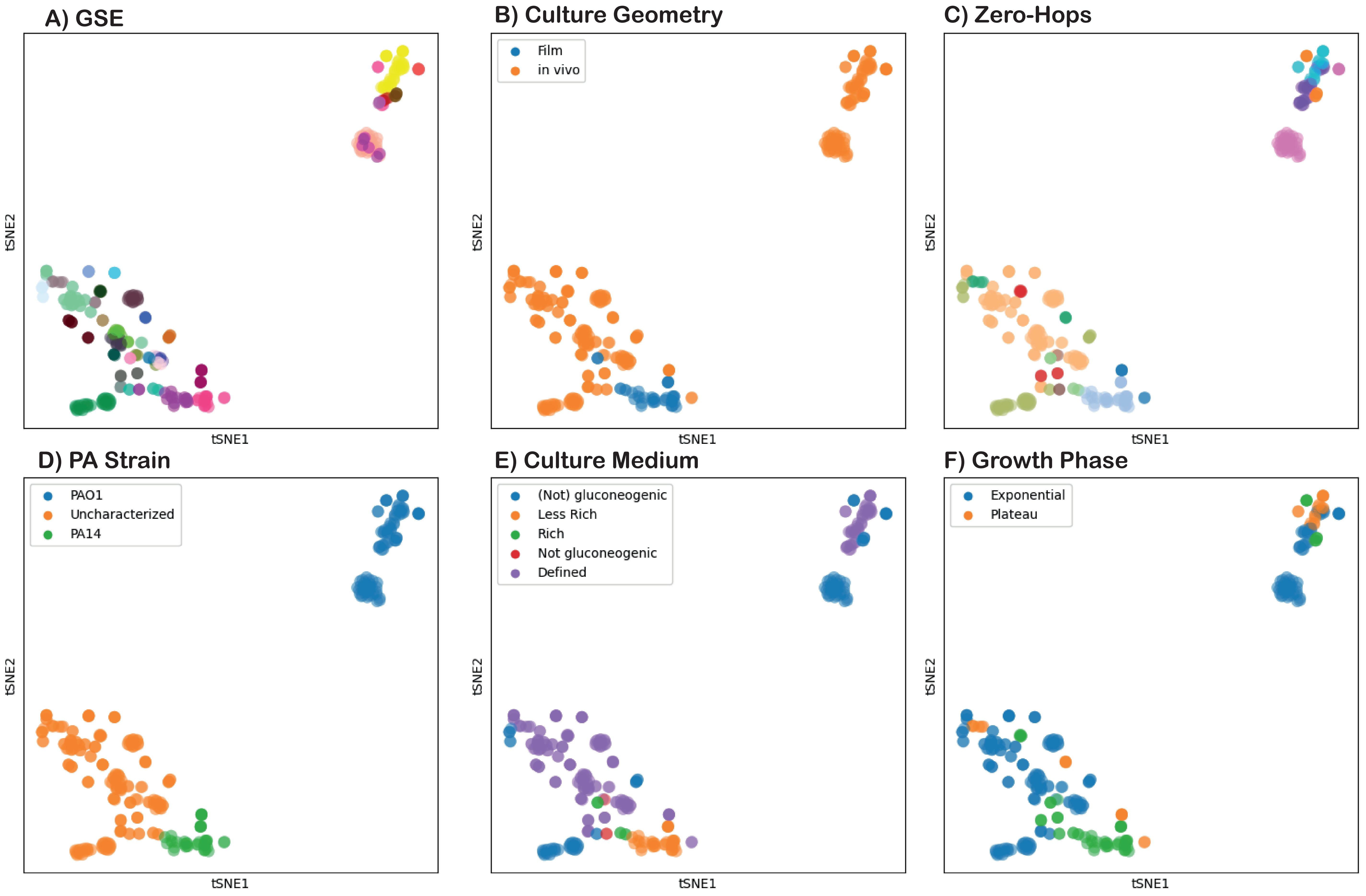
A) GSE
tSNE2
tSNE1
B) Culture Geometry
Film
in vivo
tSNE2
tSNE1
C) Zero-Hops
tSNE2
tSNE1
D) PA Strain
PAO1
Uncharacterized
PA14
tSNE2
tSNE1
E) Culture Medium
(Not) gluconeogenic
Less Rich
Rich
Not gluconeogenic
Defined
tSNE2
tSNE1
F) Growth Phase
Exponential
Plateau
tSNE2
tSNE1

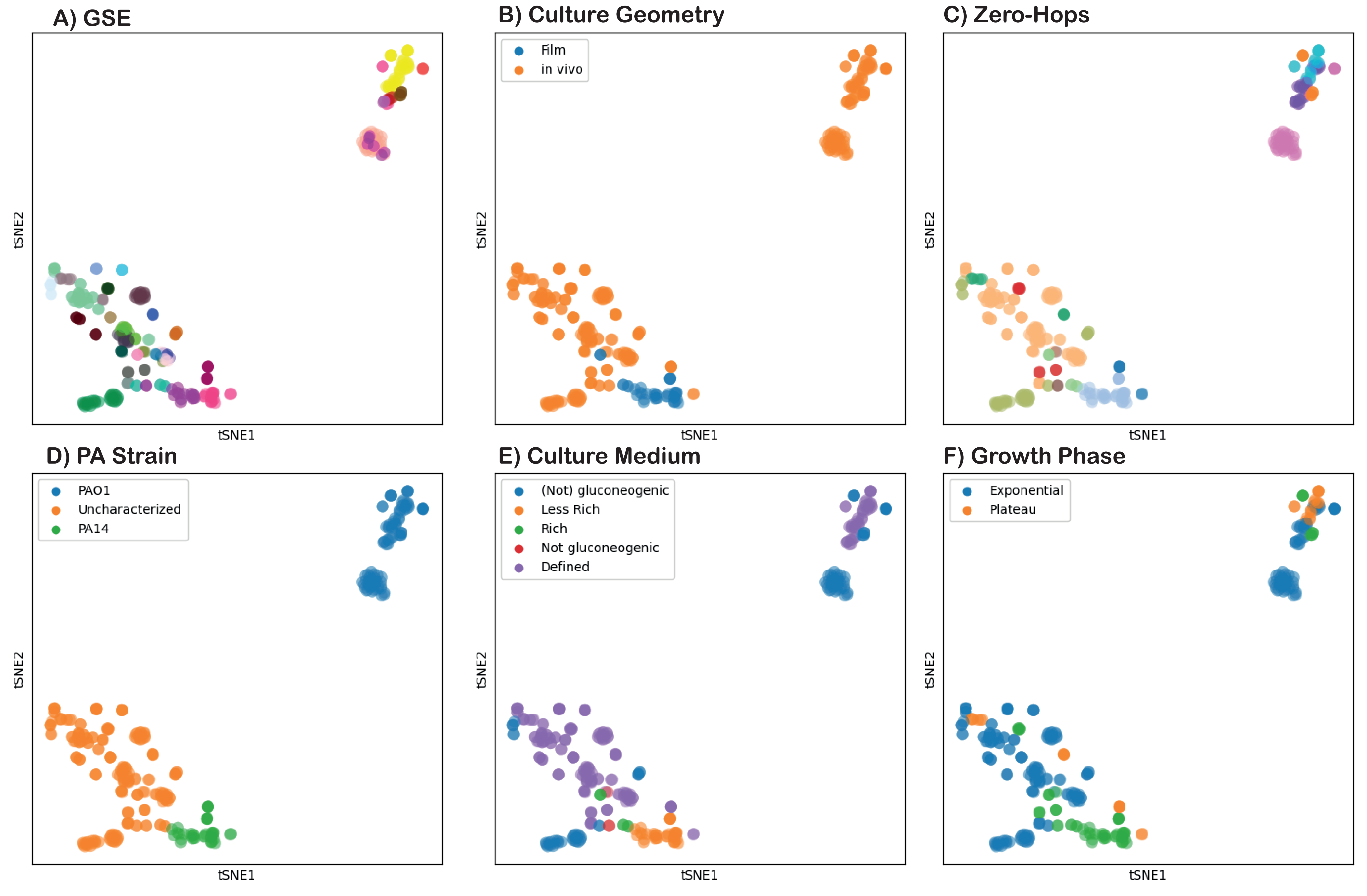
A) GSE
tSNE2
tSNE1
B) Culture Geometry
Film
in vivo
tSNE2
tSNE1
C) Zero-Hops
tSNE2
tSNE1
D) PA Strain
PAO1
Uncharacterized
PA14
tSNE2
tSNE1
E) Culture Medium
(Not) gluconeogenic
Less Rich
Rich
Not gluconeogenic
Defined
tSNE2
tSNE1
F) Growth Phase
Exponential
Plateau
tSNE2
tSNE1
