# Supplementary material for: reComBat: batch-effect removal in large-scale multi-source gene-expression data integration
Source: Bioinform Adv. 2022 Oct 6;2(1):vbac071. doi: 10.1093/bioadv/vbac071 (PMC9710604; doi:10.1093/bioadv/vbac071)
Supplement: vbac071_Supplementary_Data [file vbac071_supplementary_data.zip › vbac071_supplement/supplFigures/Raw.pdf]

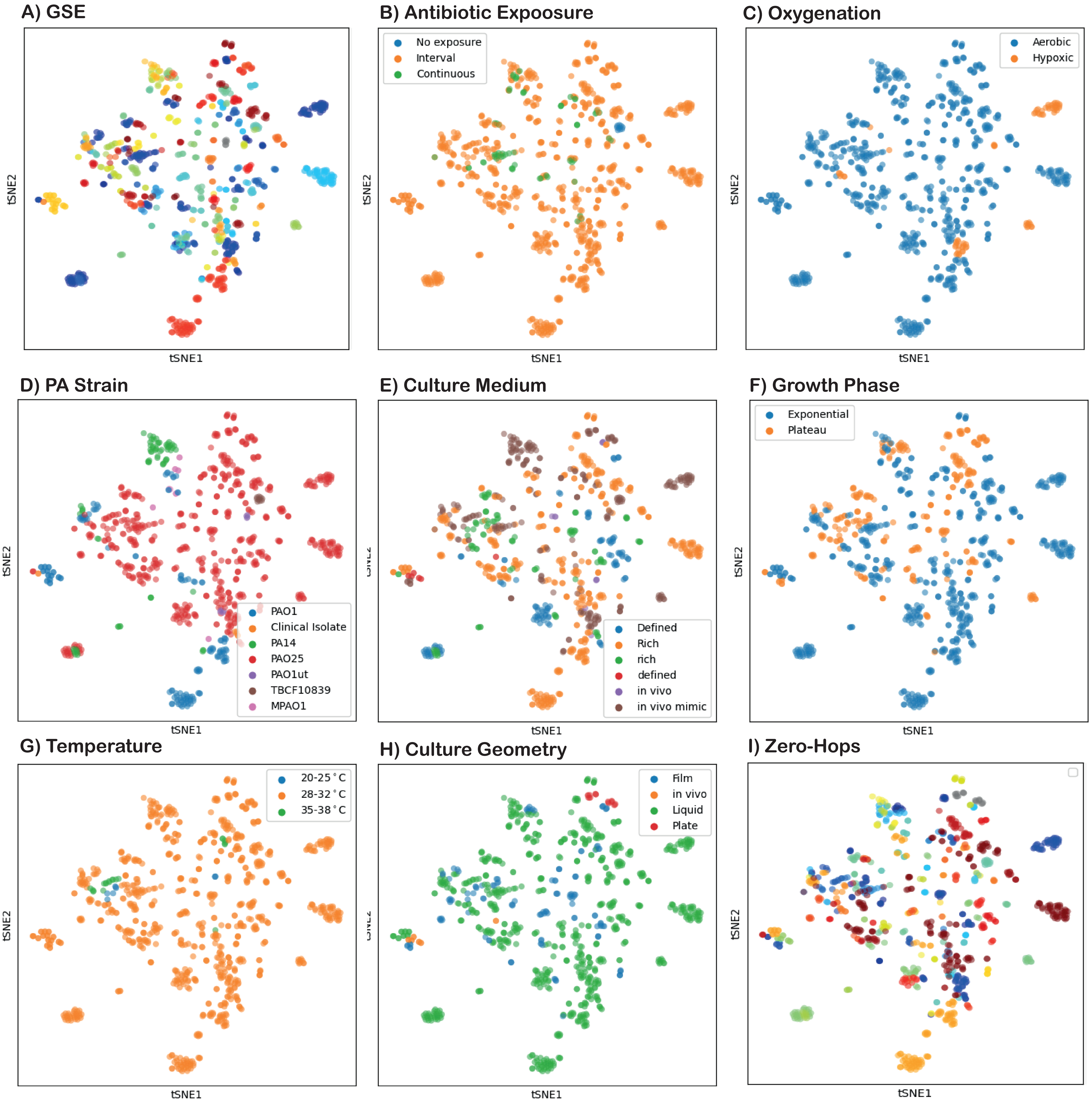
A) GSE
tSNE2
tSNE1
B) Antibiotic Expoosure
No exposure
Interval
Continuous
tSNE2
tSNE1
C) Oxygenation
Aerobic
Hypoxic
tSNE2
tSNE1
D) PA Strain
PAO1
Clinical Isolate
PA14
PAO25
PAO1ut
TBCF10839
MPAO1
tSNE2
tSNE1
E) Culture Medium
Defined
Rich
rich
defined
in vivo
in vivo mimic
tSNE1
F) Growth Phase
Exponential
Plateau
tSNE2
tSNE1
G) Temperature
20-25 °C
28-32 °C
35-38 °C
tSNE2
tSNE1
H) Culture Geometry
Film
in vivo
Liquid
Plate
tSNE1
I) Zero-Hops
tSNE2
tSNE1

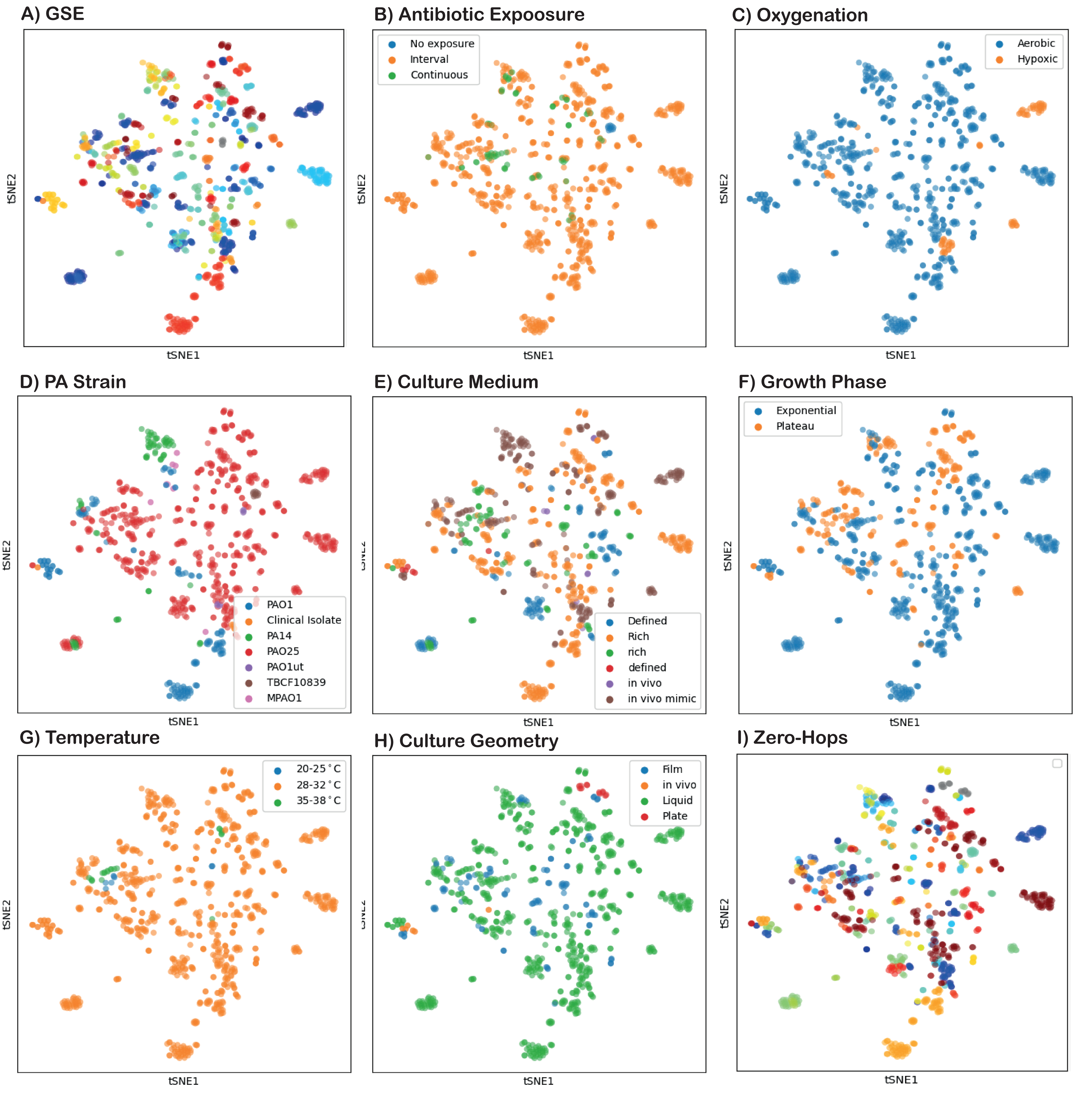
A) GSE
B) Antibiotic Expoosure
No exposure
Interval
Continuous
C) Oxygenation
Aerobic
Hypoxic
D) PA Strain
PAO1
Clinical Isolate
PA14
PAO25
PAO1ut
TBCF10839
MPAO1
E) Culture Medium
Defined
Rich
rich
defined
in vivo
in vivo mimic
F) Growth Phase
Exponential
Plateau
G) Temperature
20-25 °C
28-32 °C
35-38 °C
H) Culture Geometry
Film
in vivo
Liquid
Plate
I) Zero-Hops
tSNE1
tSNE2
