# Supplementary material for: reComBat: batch-effect removal in large-scale multi-source gene-expression data integration
Source: Bioinform Adv. 2022 Oct 6;2(1):vbac071. doi: 10.1093/bioadv/vbac071 (PMC9710604; doi:10.1093/bioadv/vbac071)
Supplement: vbac071_Supplementary_Data [file vbac071_supplementary_data.zip › vbac071_supplement/supplFigures/standardised_RNA.pdf]

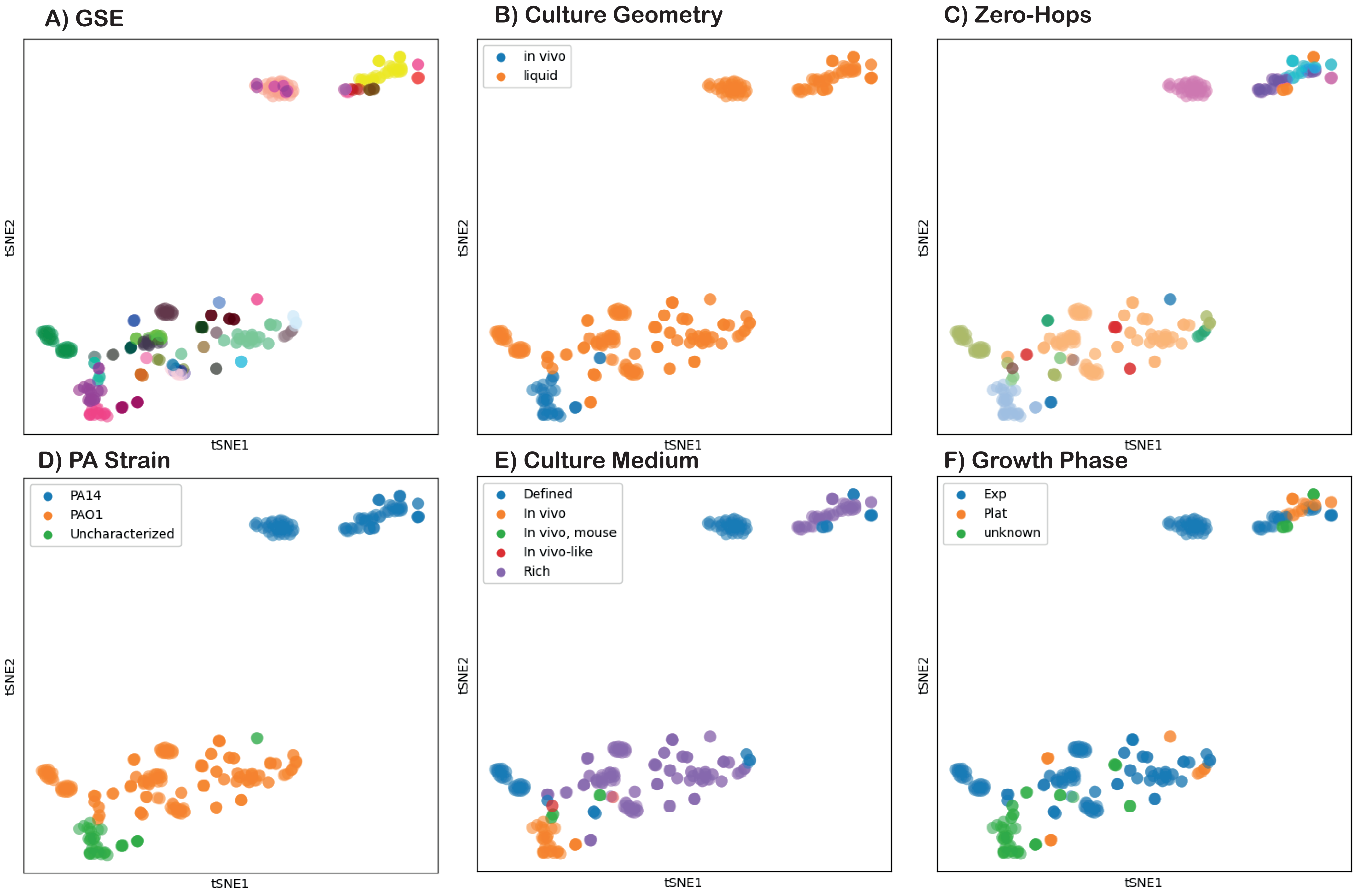

A) GSE
B) Culture Geometry
in vivo
liquid
C) Zero-Hops
D) PA Strain
PA14
PAO1
Uncharacterized
E) Culture Medium
Defined
In vivo
In vivo, mouse
In vivo-like
Rich
F) Growth Phase
Exp
Plat
unknown
tSNE1
tSNE2

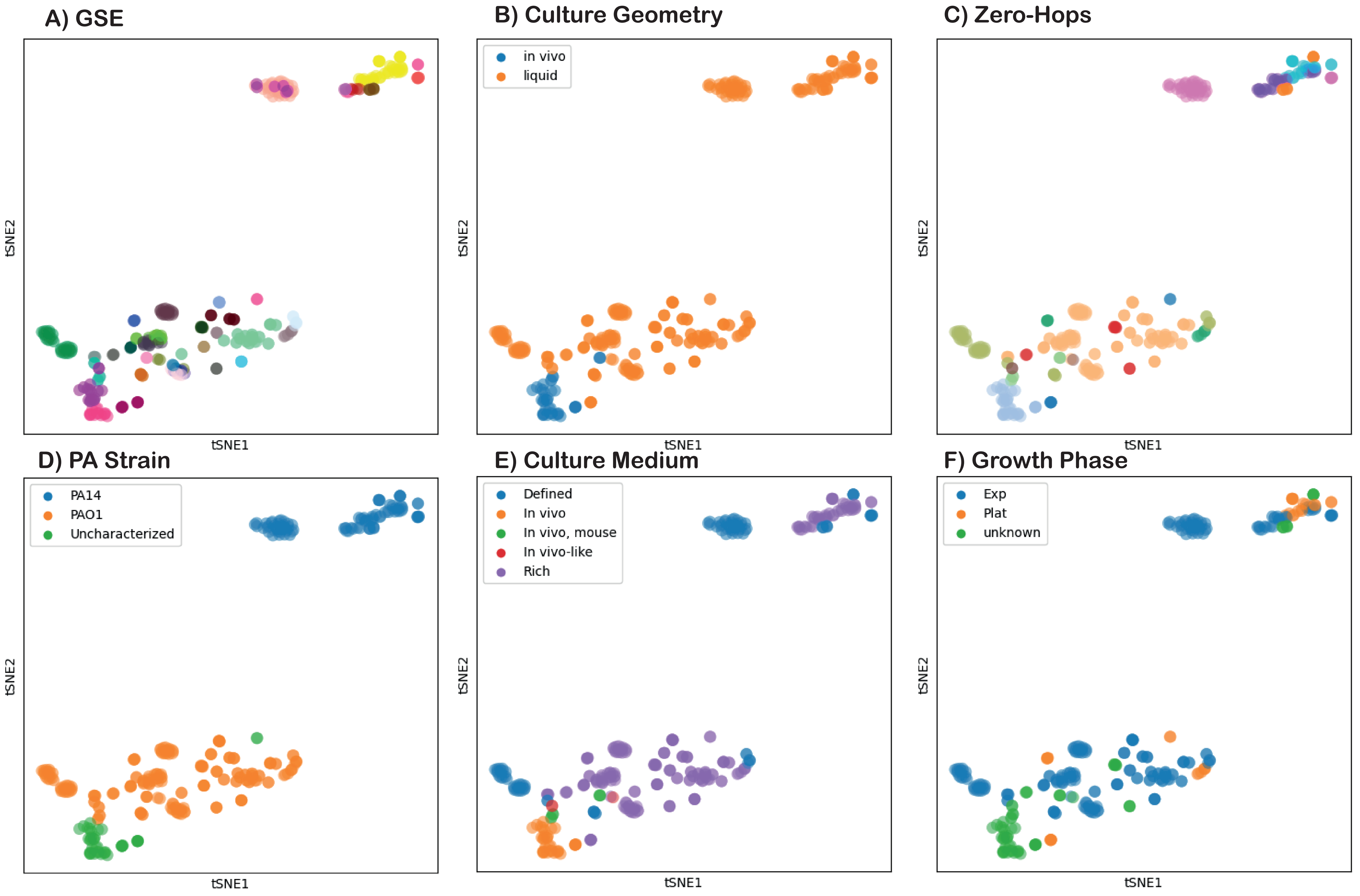
A) GSE
B) Culture Geometry
in vivo
liquid
C) Zero-Hops
D) PA Strain
PA14
PAO1
Uncharacterized
E) Culture Medium
Defined
In vivo
In vivo, mouse
In vivo-like
Rich
F) Growth Phase
Exp
Plat
unknown
tSNE1
tSNE2

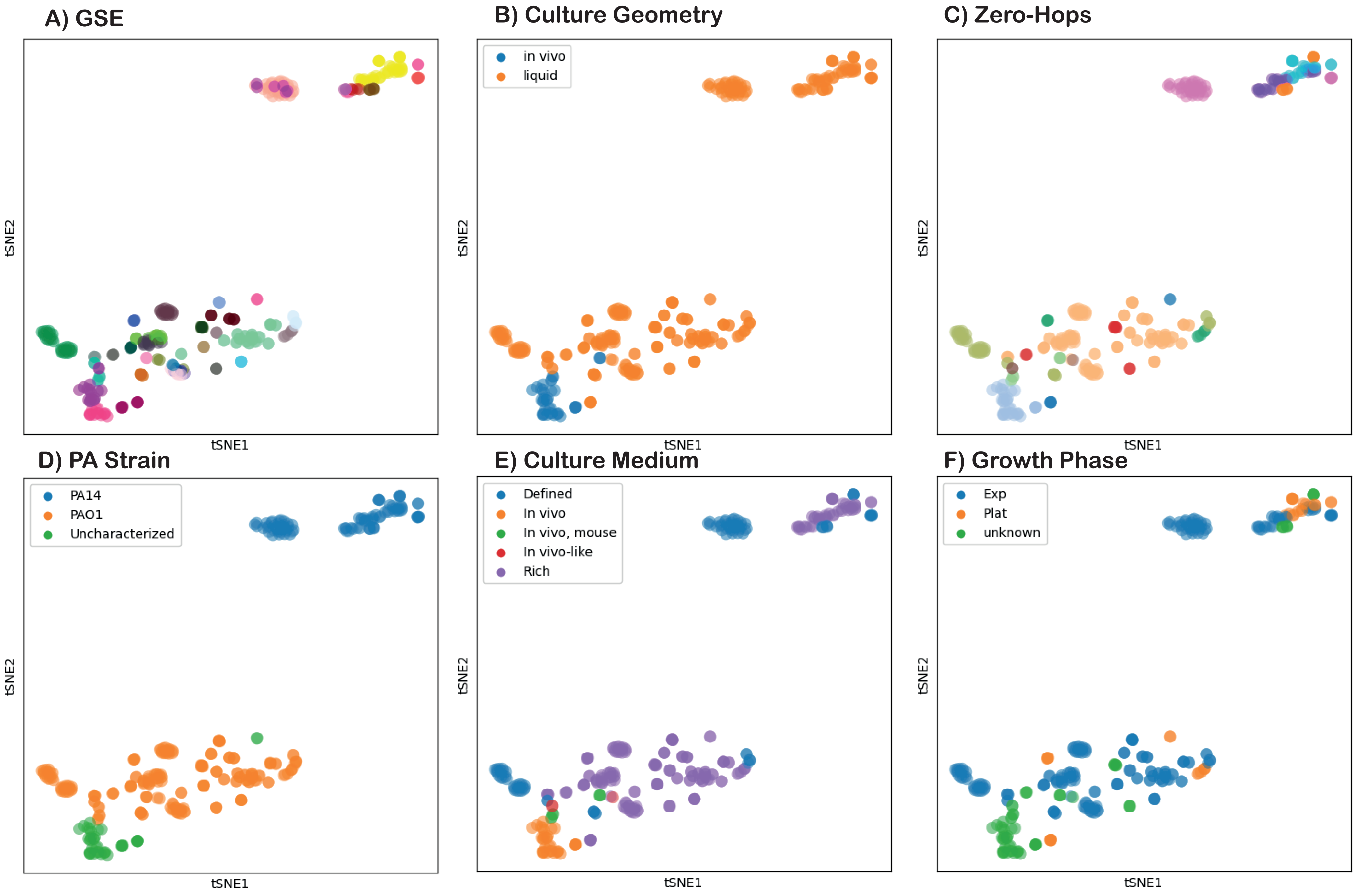
A) GSE
B) Culture Geometry
in vivo
liquid
C) Zero-Hops
D) PA Strain
PA14
PAO1
Uncharacterized
E) Culture Medium
Defined
In vivo
In vivo, mouse
In vivo-like
Rich
F) Growth Phase
Exp
Plat
unknown
tSNE1
tSNE2
