# Supplementary figures and images for: reComBat: batch-effect removal in large-scale multi-source gene-expression data integration
Source: Bioinform Adv. 2022 Oct 6;2(1):vbac071. doi: 10.1093/bioadv/vbac071 (PMC9710604; doi:10.1093/bioadv/vbac071)

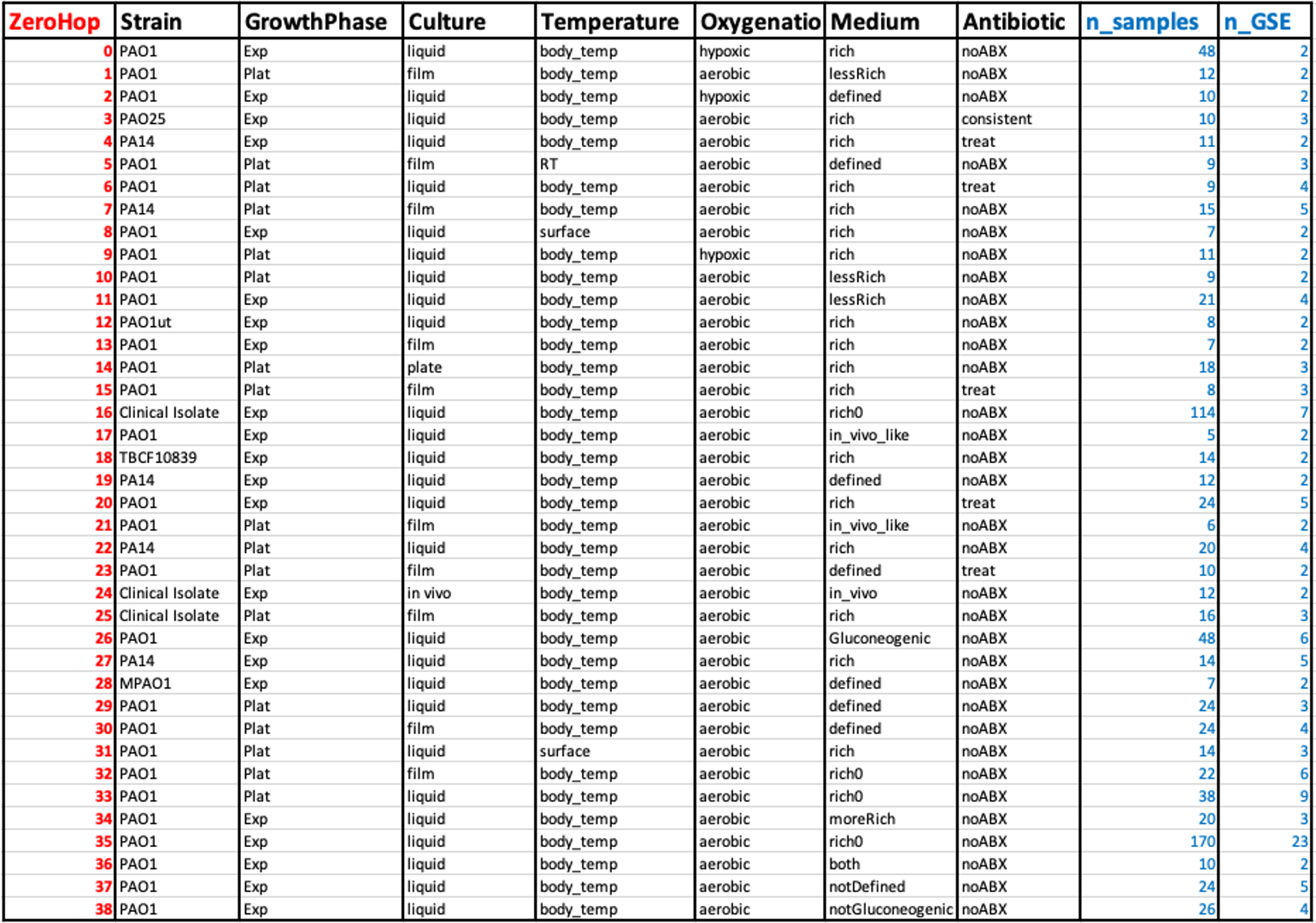

Supplement: vbac071_Supplementary_Data [file vbac071_supplementary_data.zip › vbac071_supplement/supplFigures/allZHs.png]

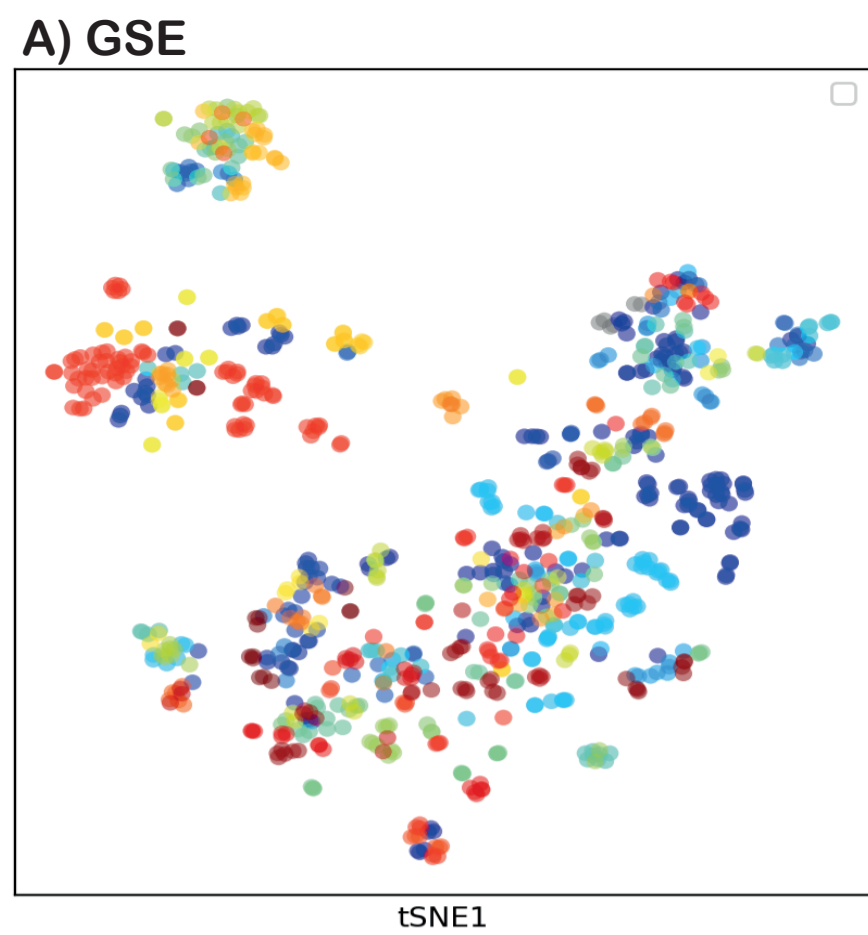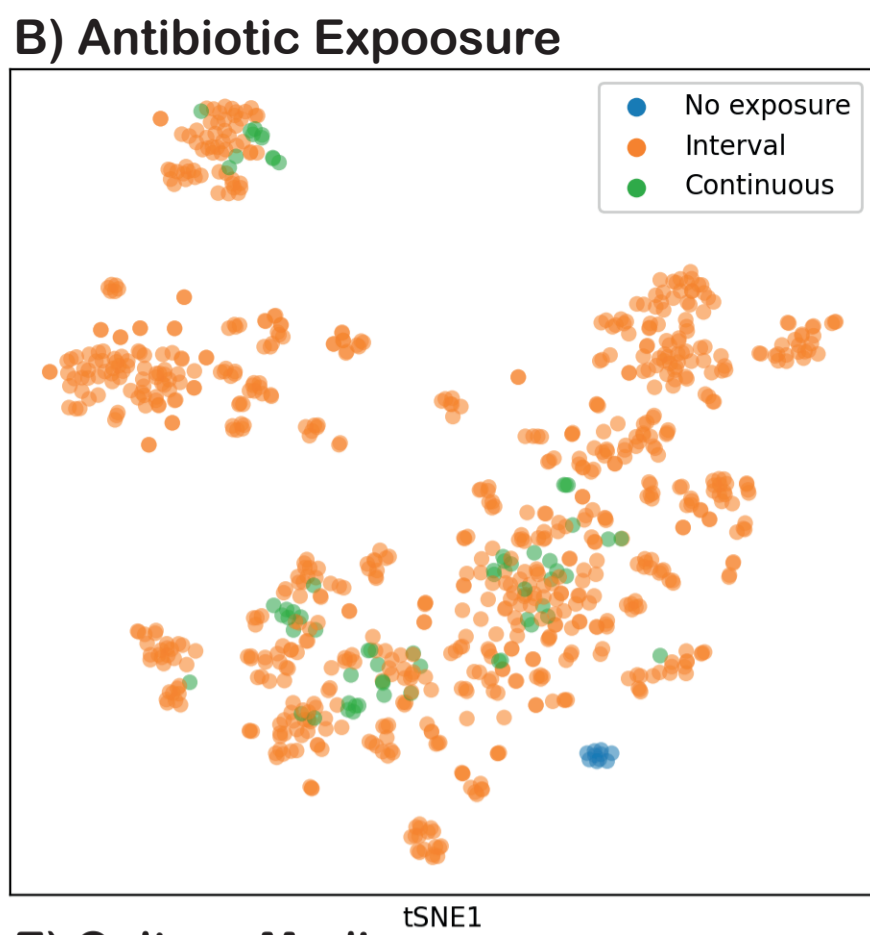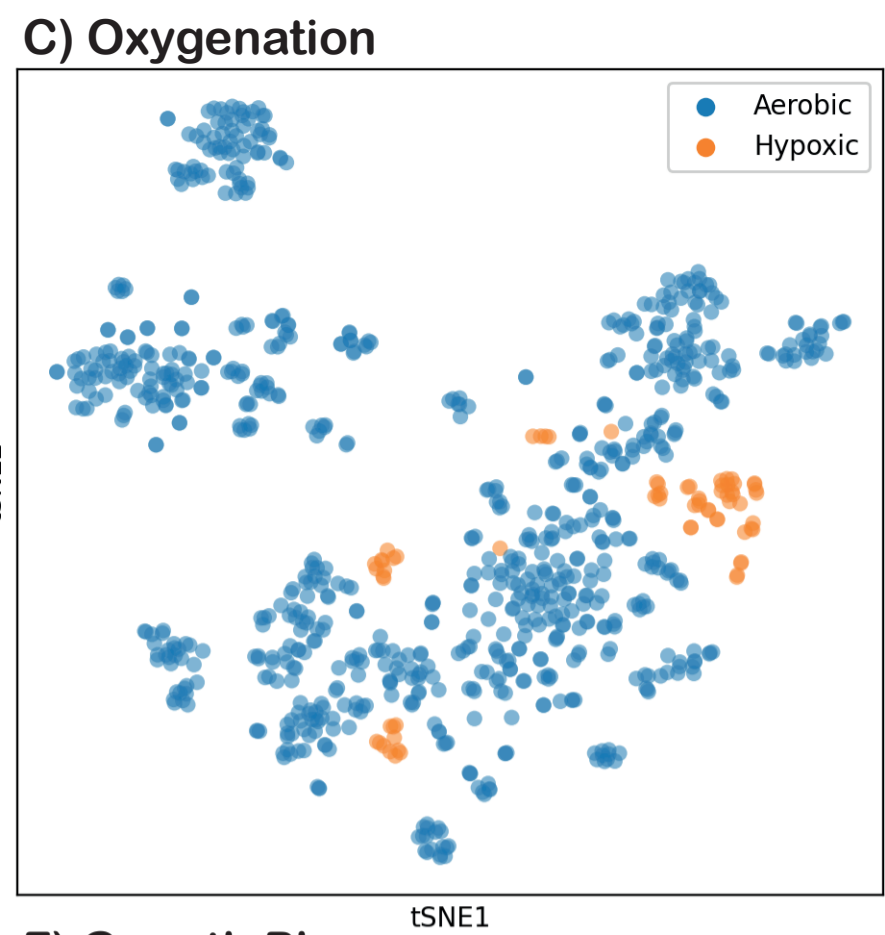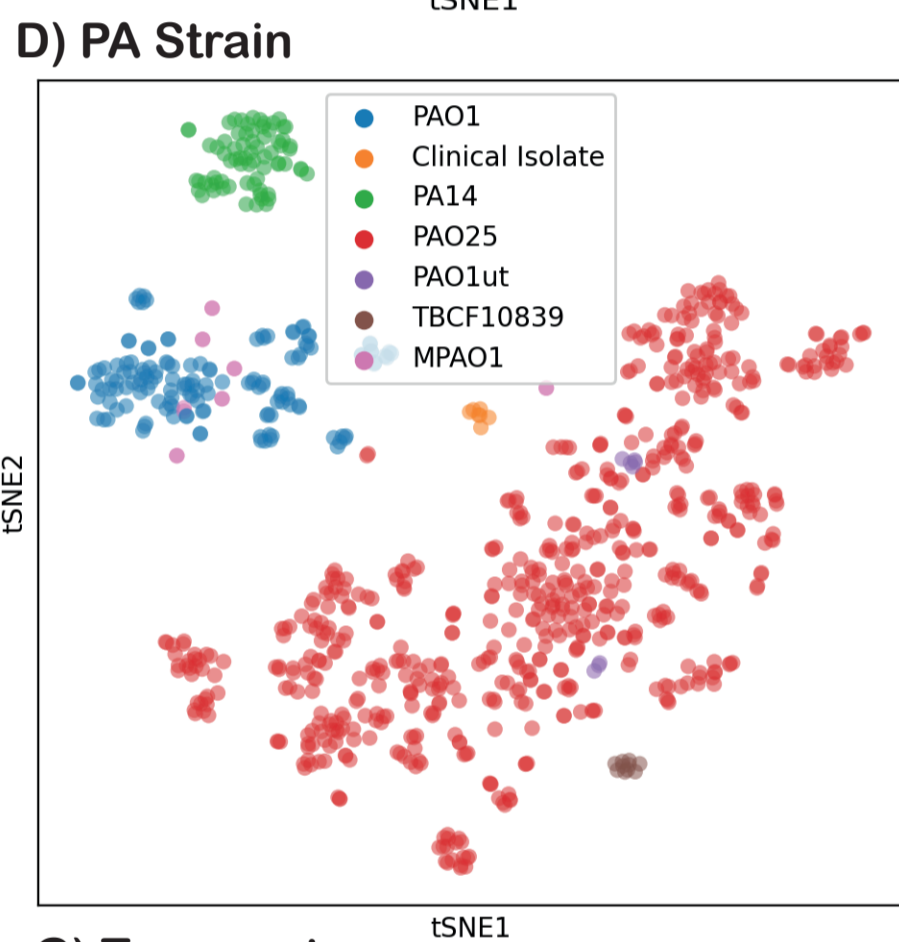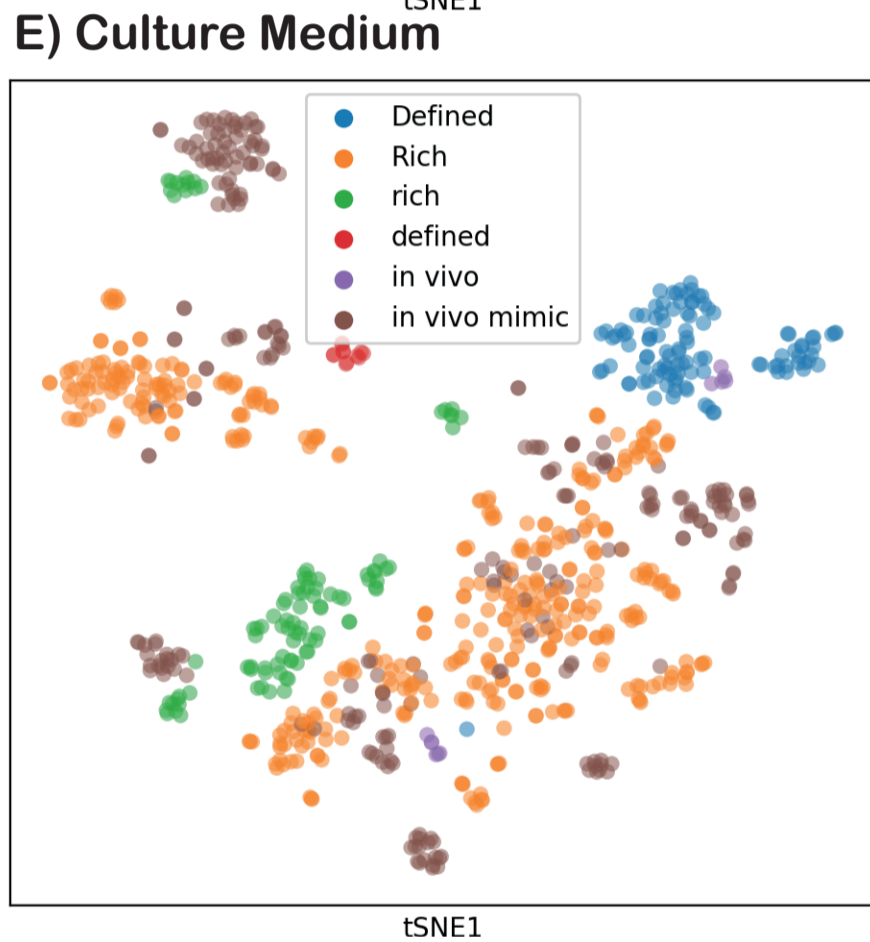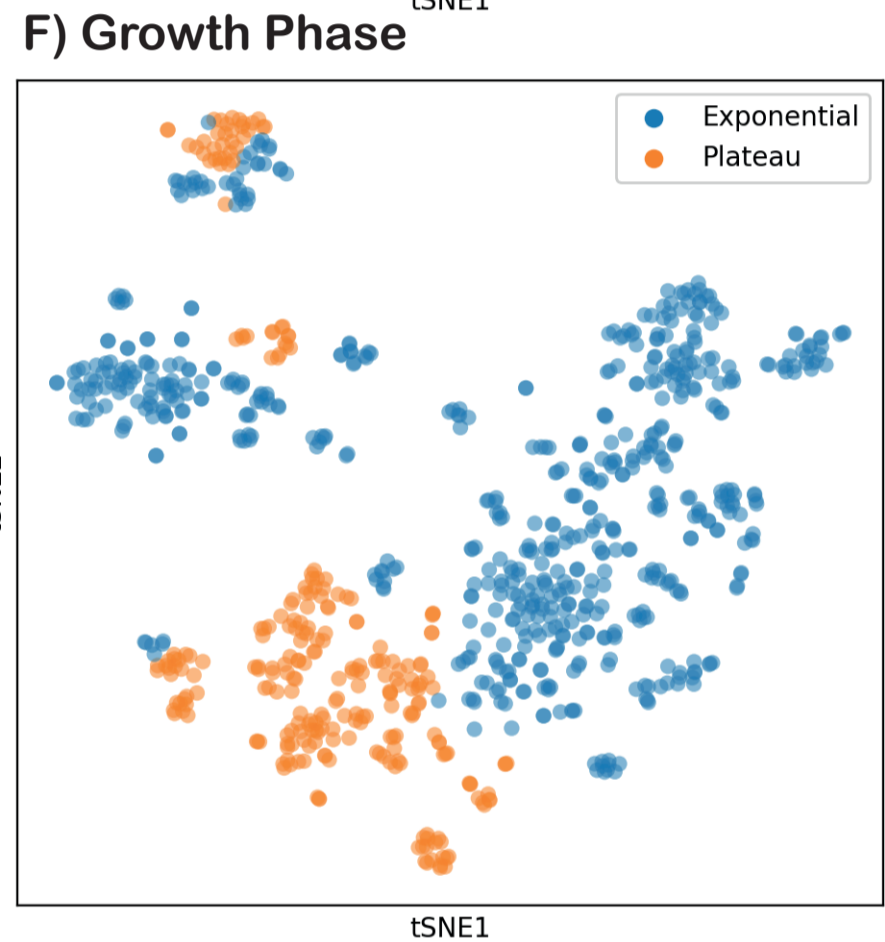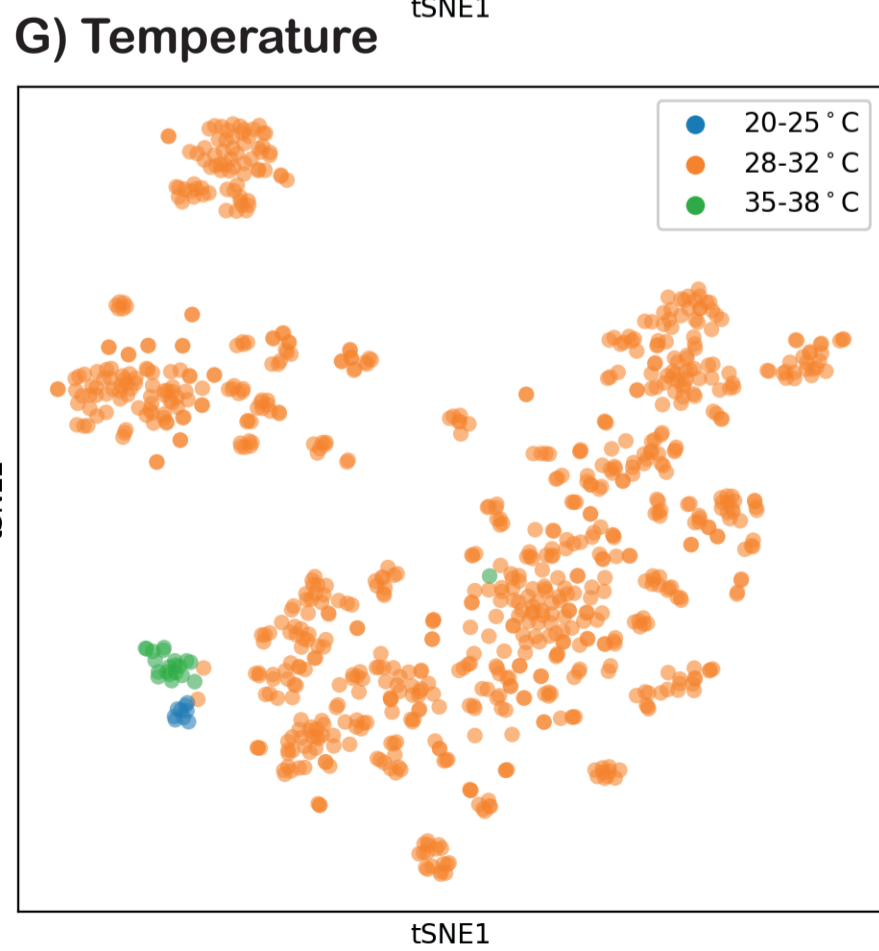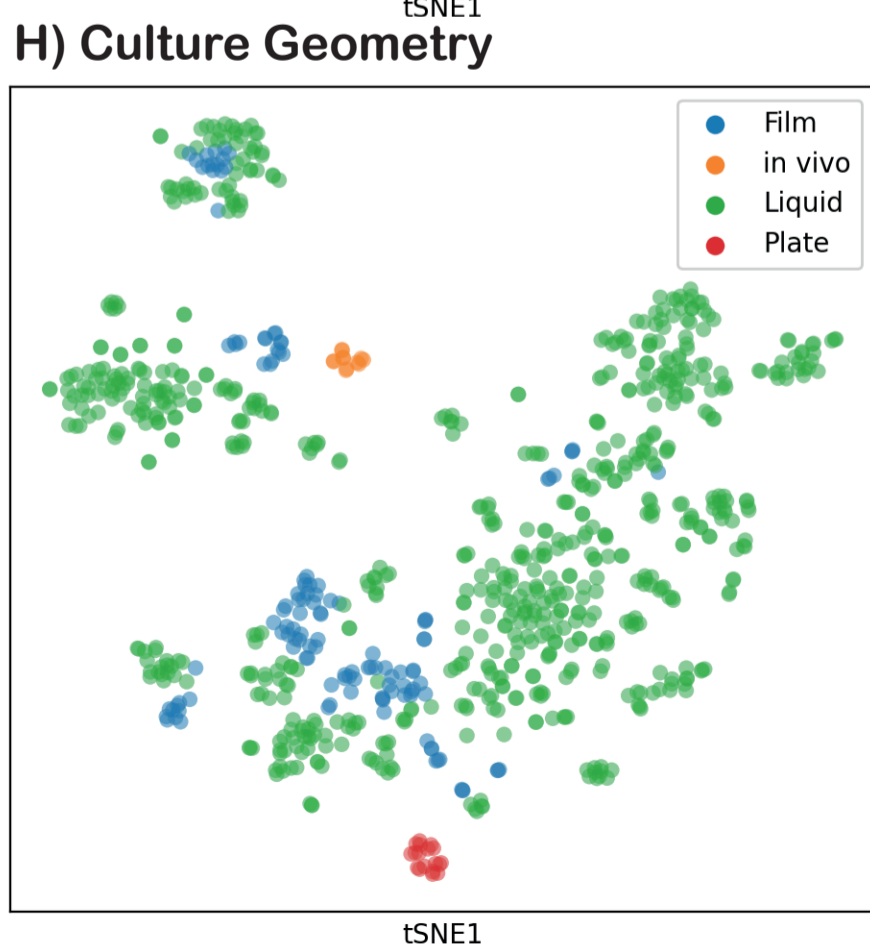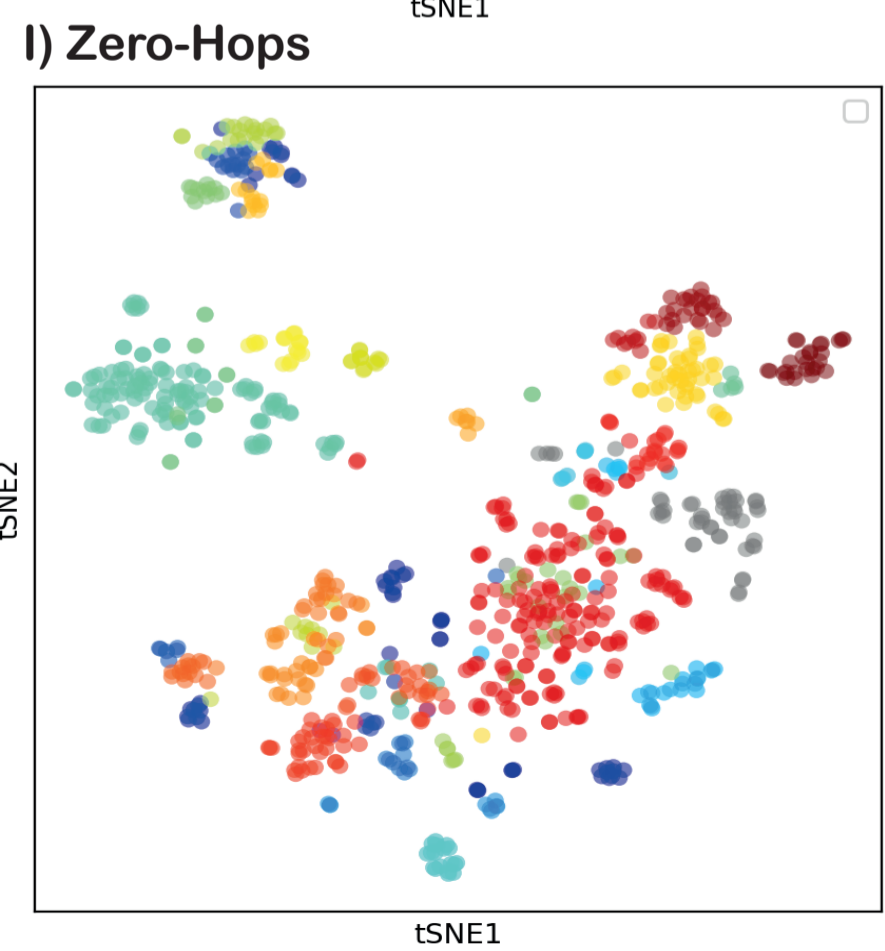

Supplement: vbac071_Supplementary_Data [file vbac071_supplementary_data.zip › vbac071_supplement/supplFigures/Combat_1.pdf]

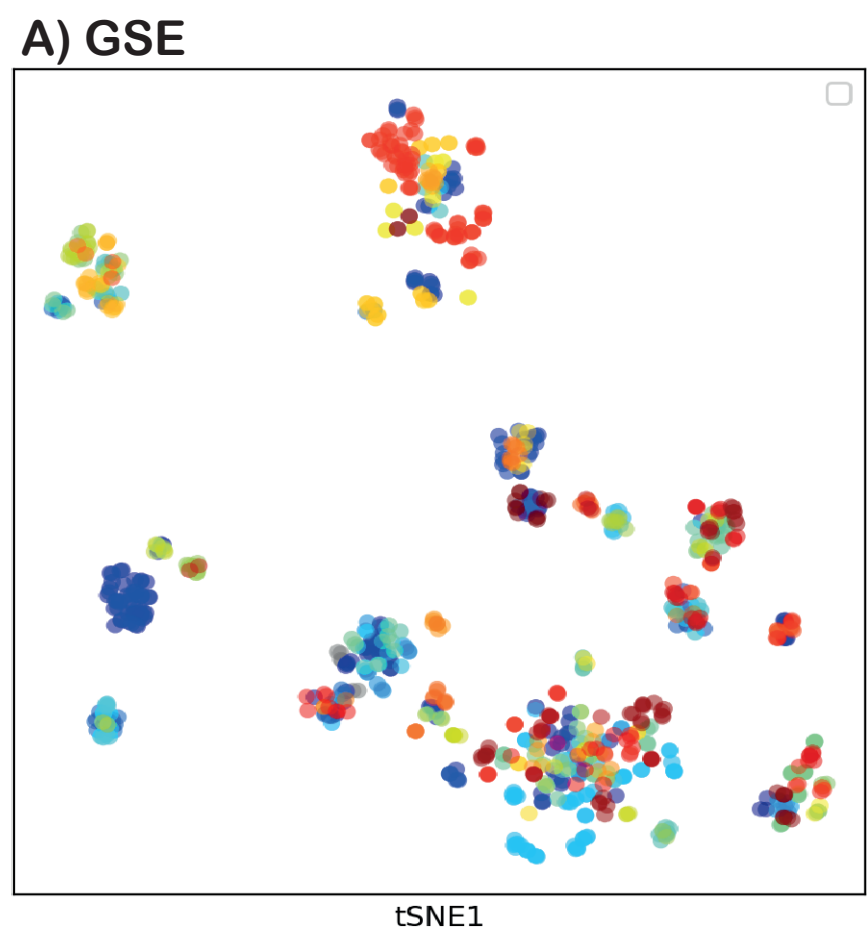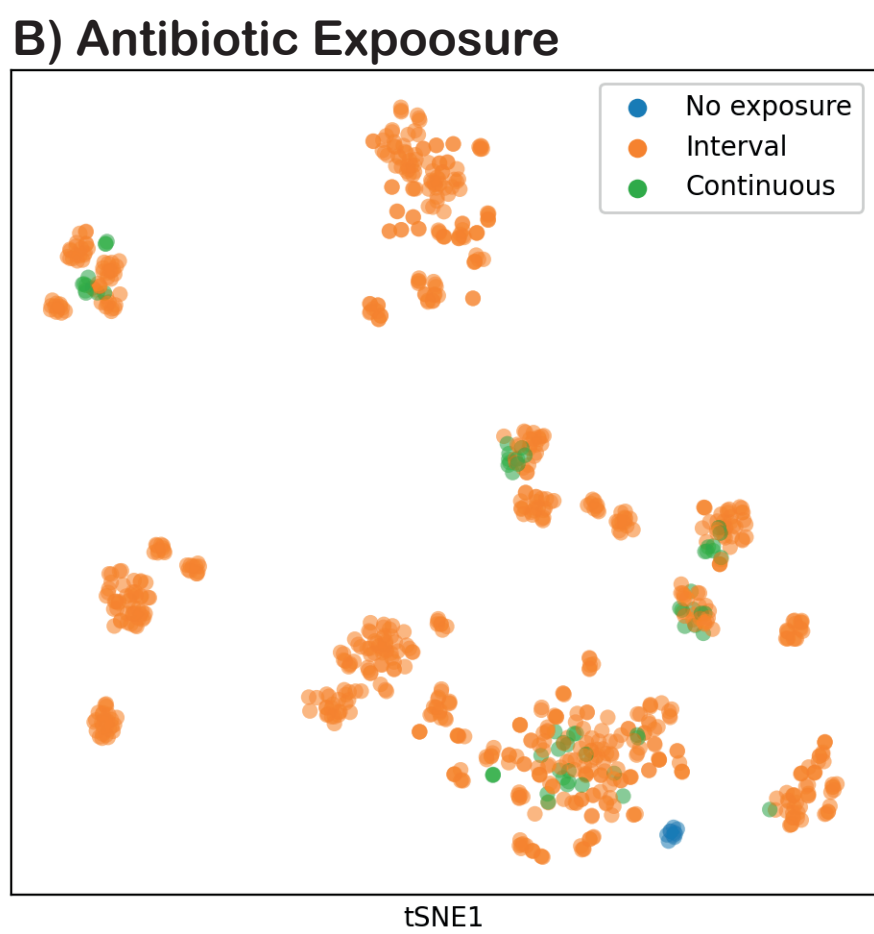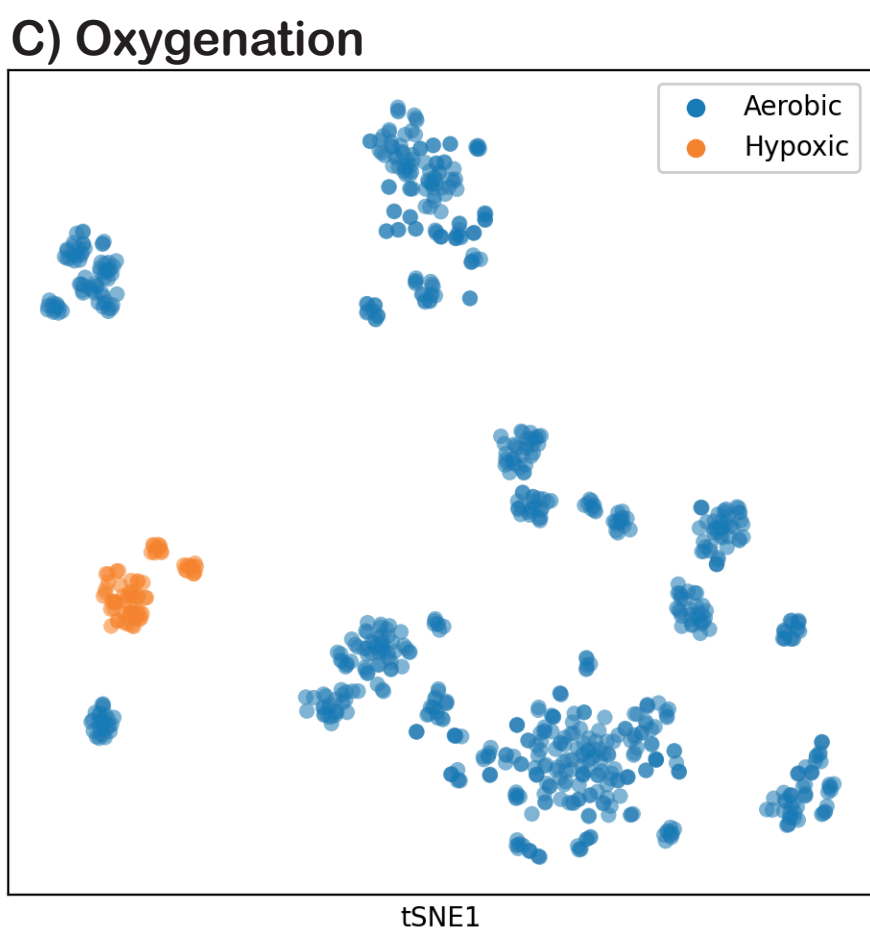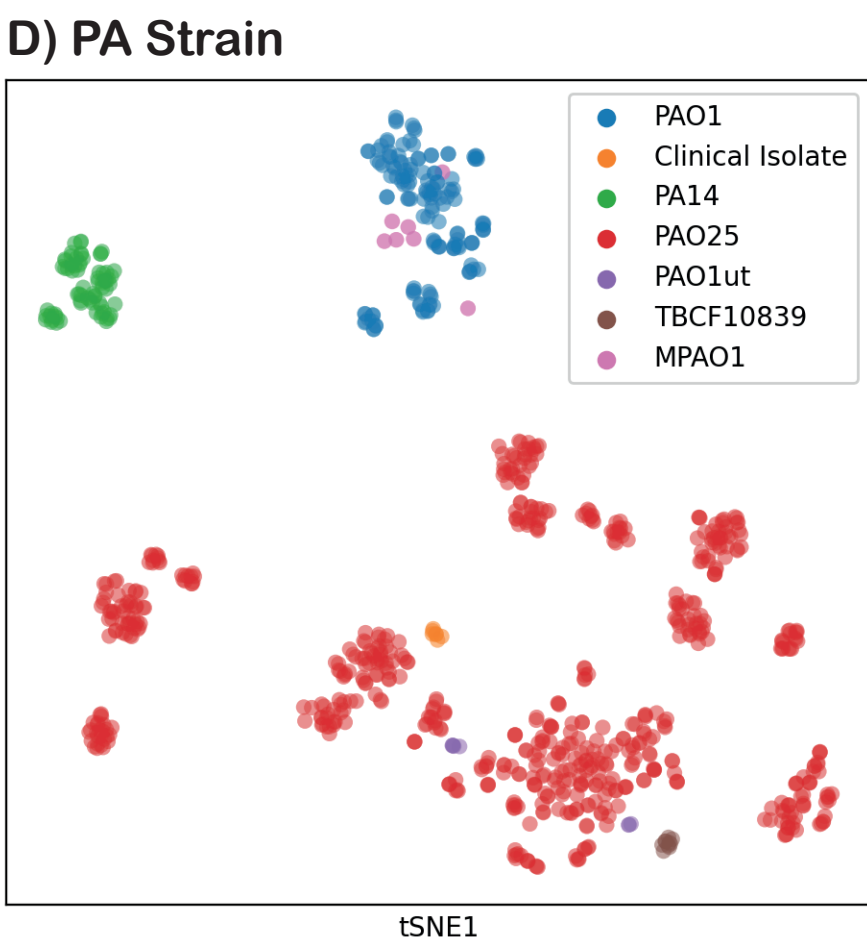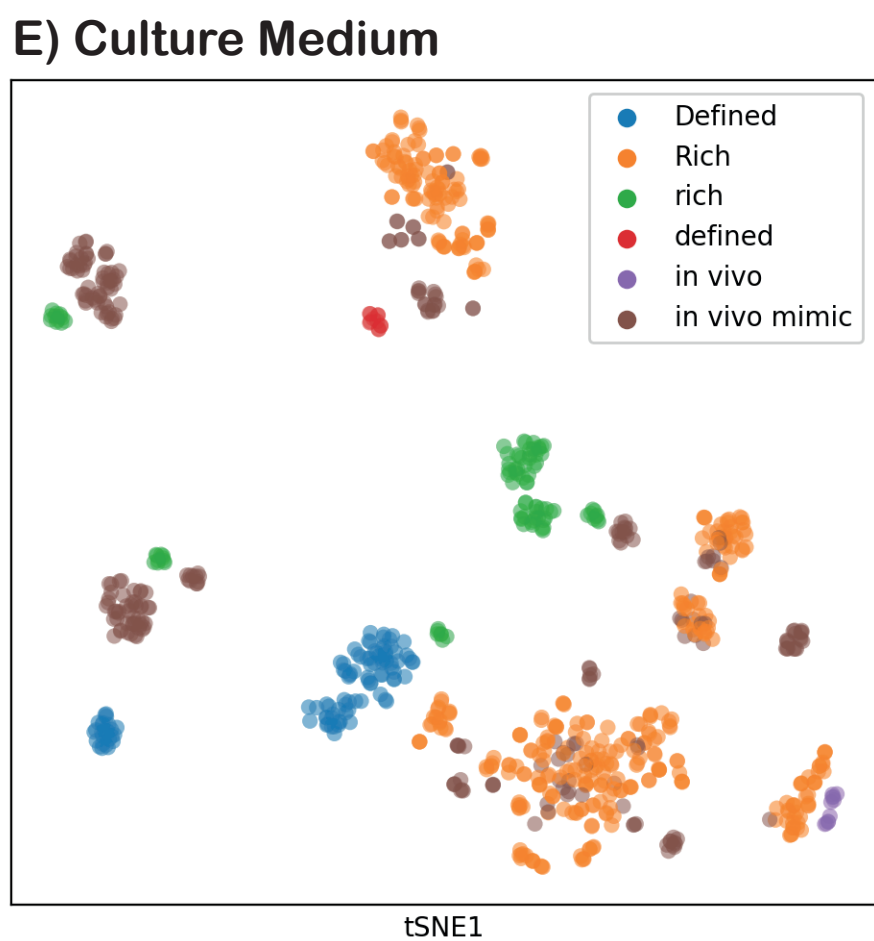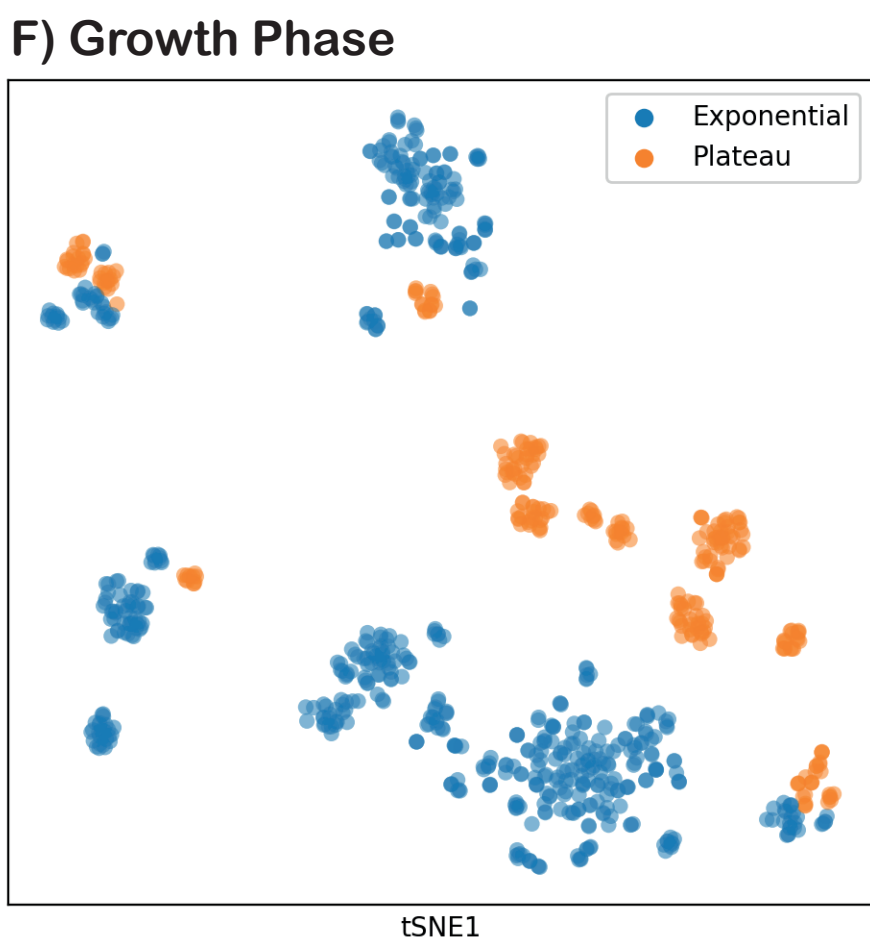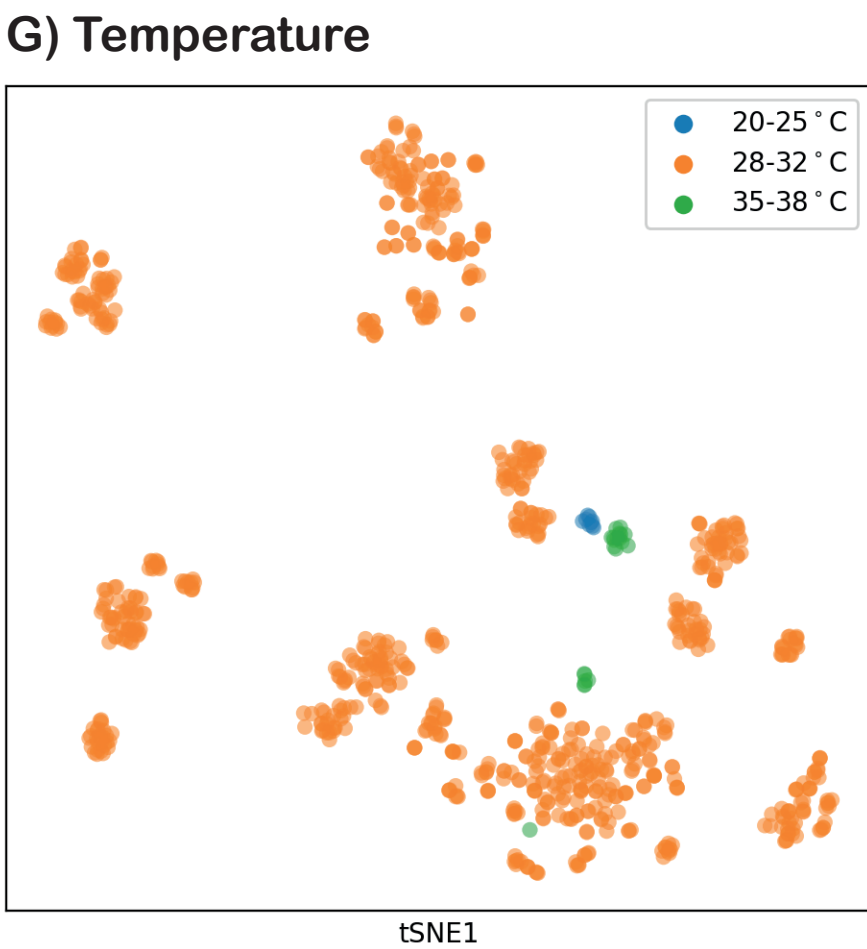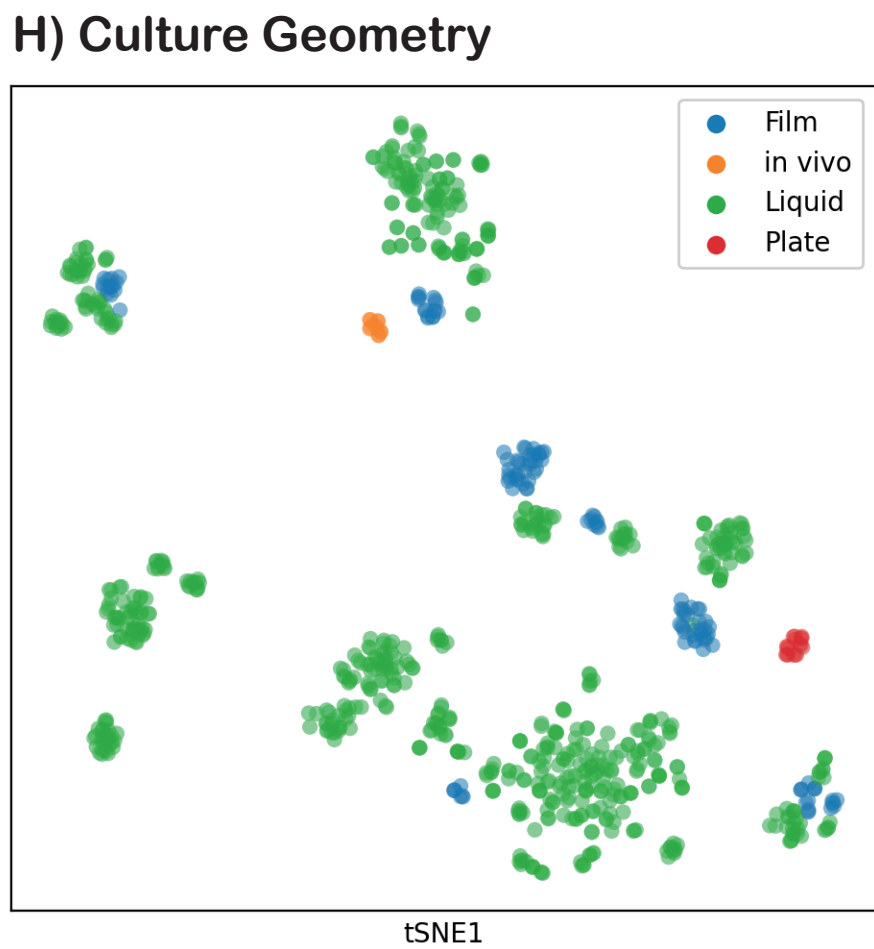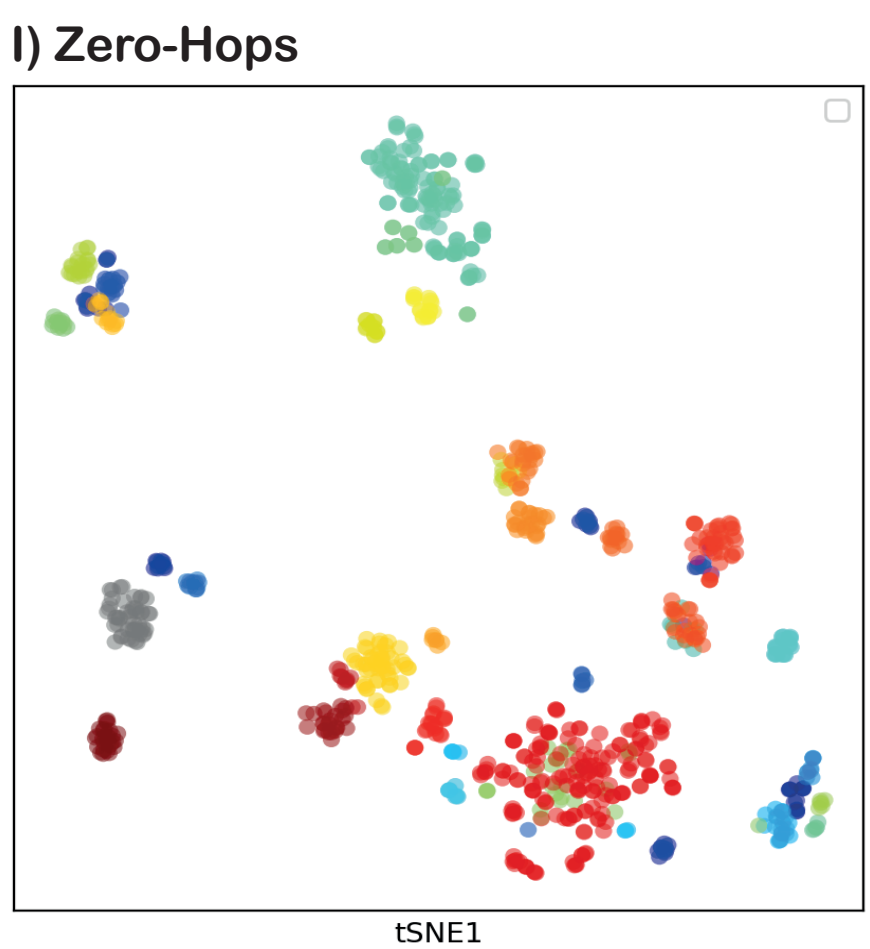

Supplement: vbac071_Supplementary_Data [file vbac071_supplementary_data.zip › vbac071_supplement/supplFigures/Combat_LASSO.pdf]

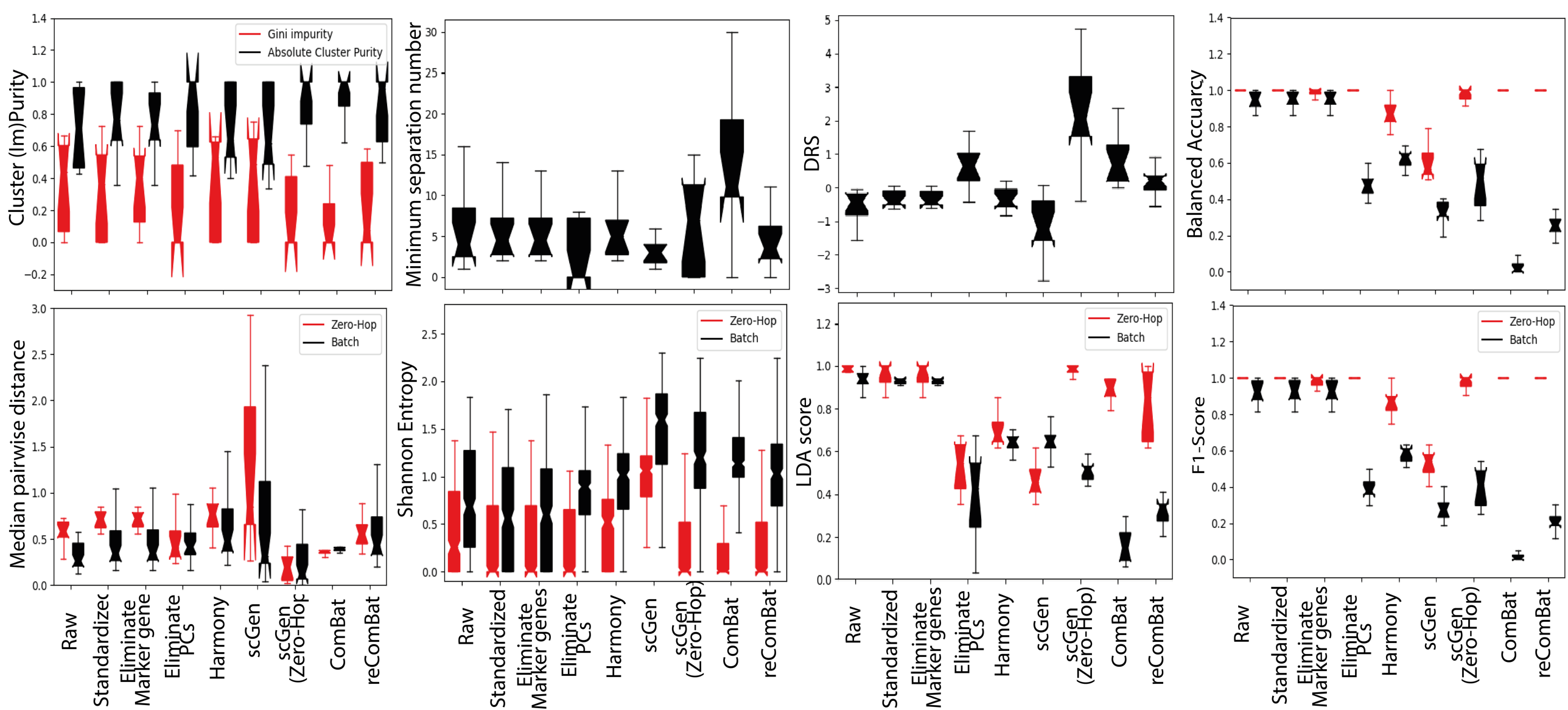

Supplement: vbac071_Supplementary_Data [file vbac071_supplementary_data.zip › vbac071_supplement/supplFigures/MA_eval_RNA.png]

A) GSE

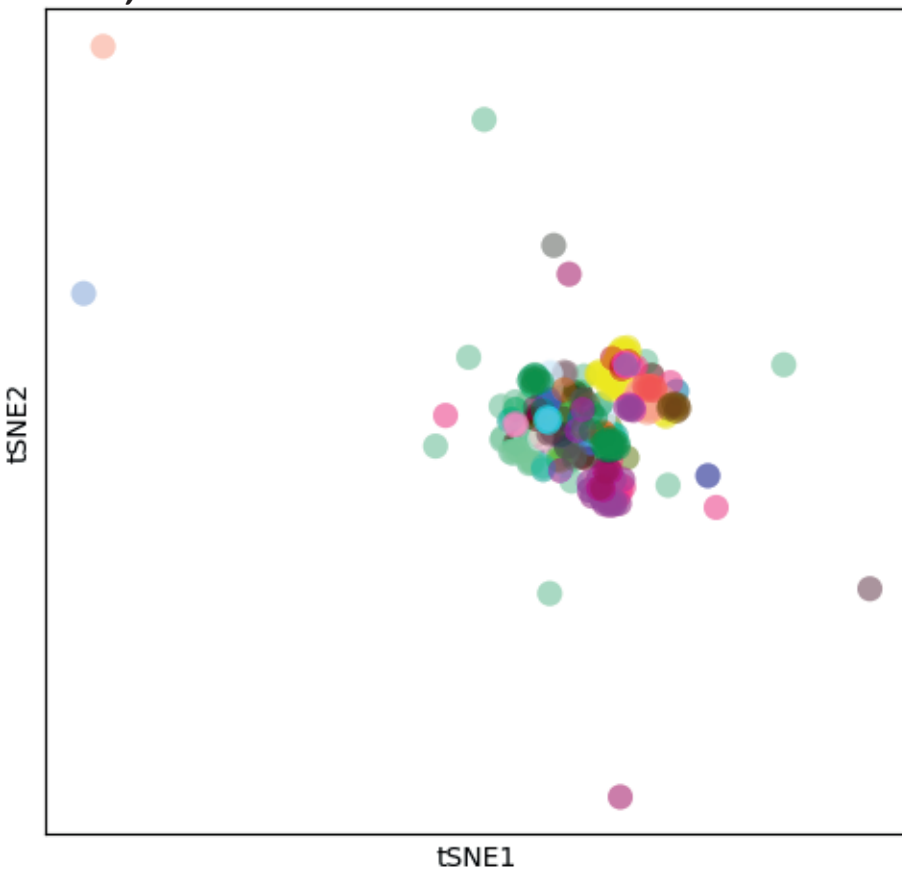

B) Culture Geometry

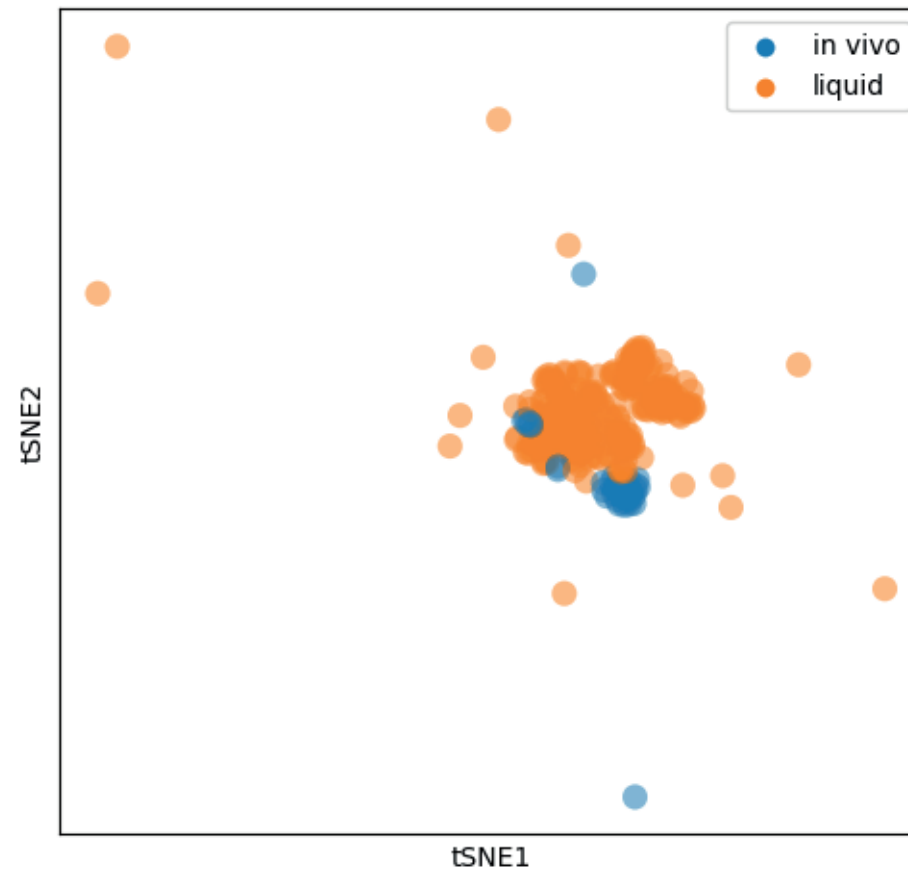

C) Zero-Hops

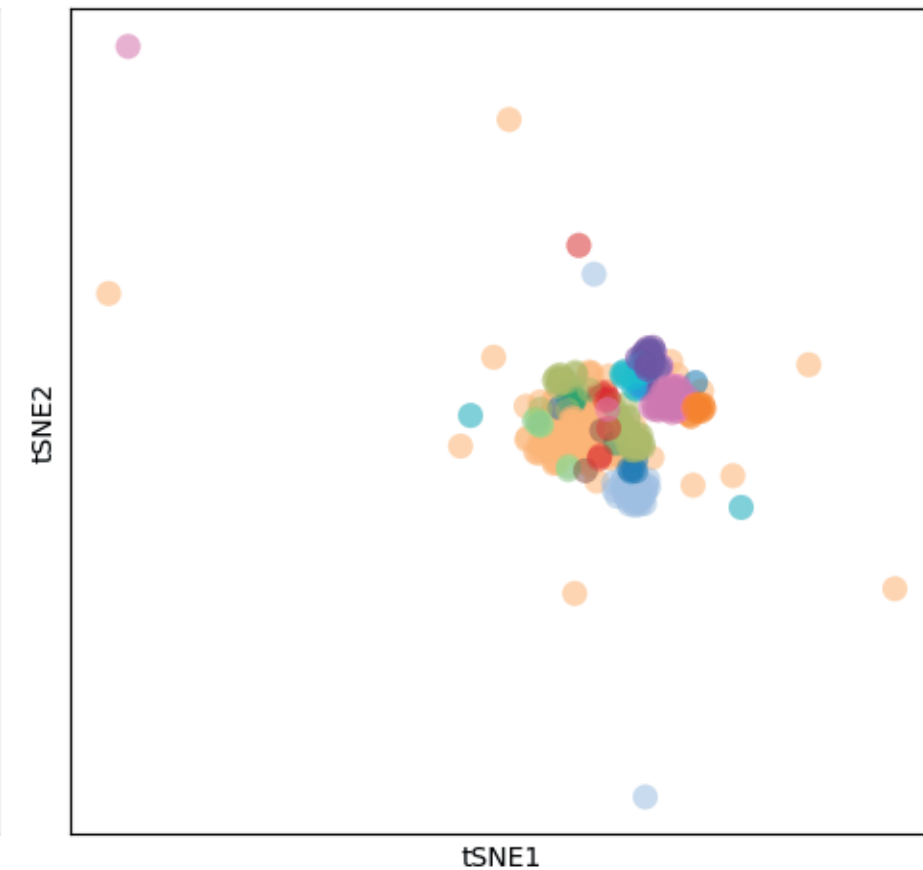

D) PA Strain

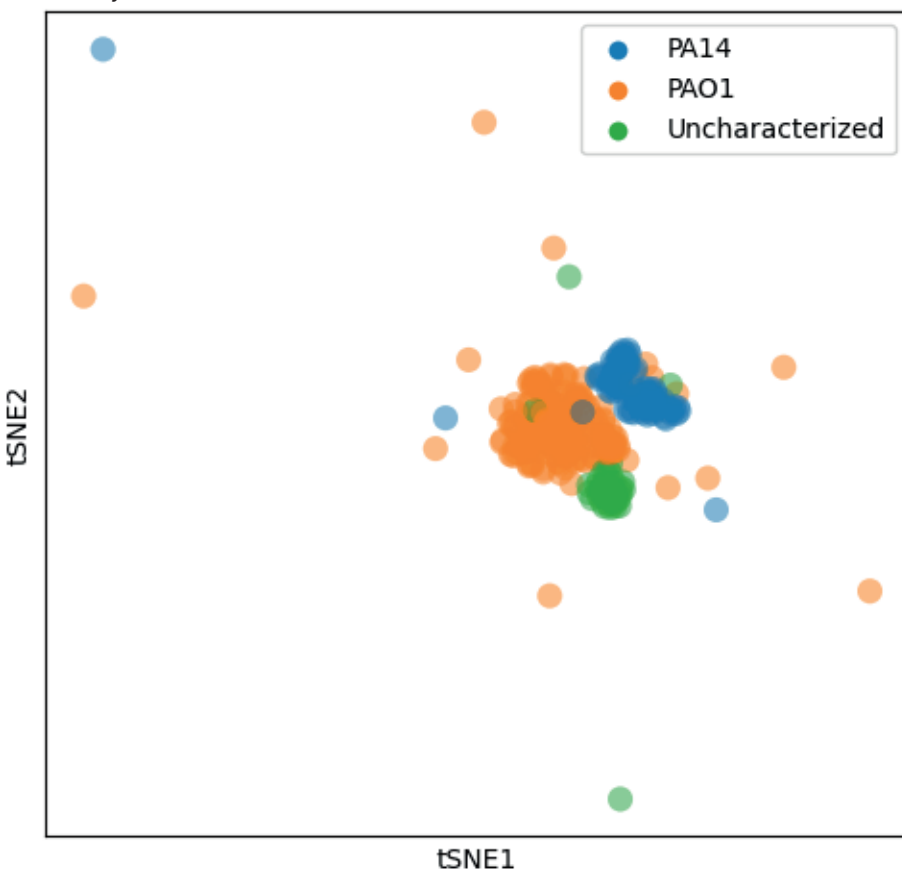

E) Culture Medium

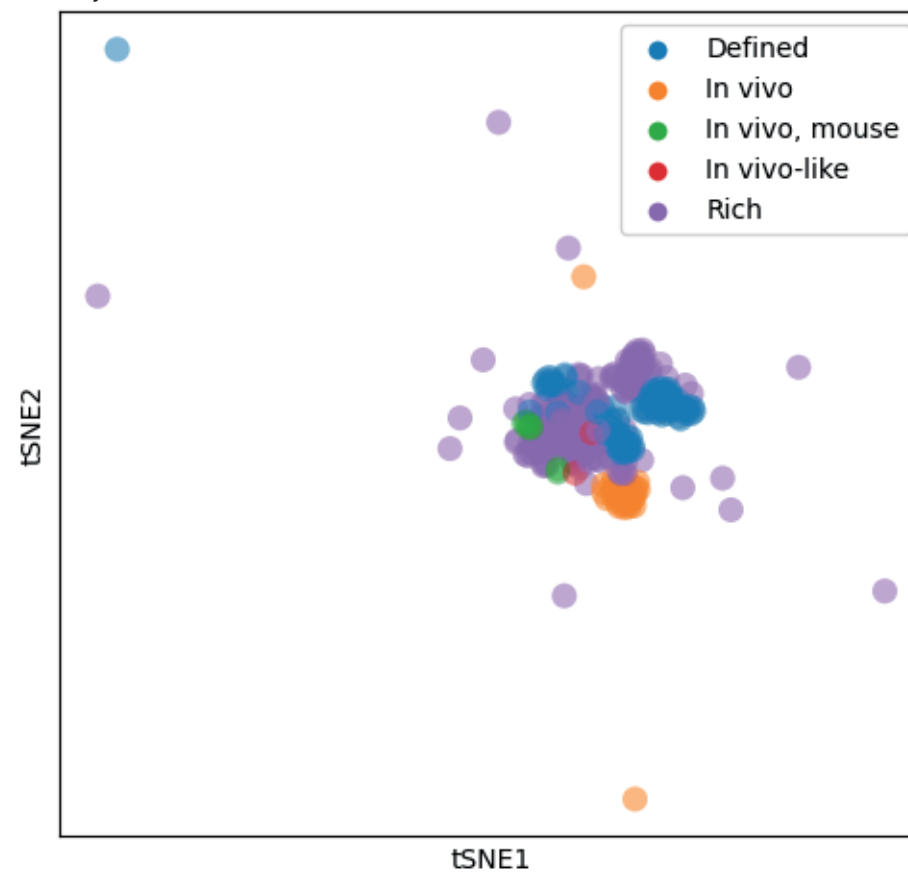

F) Growth Phase

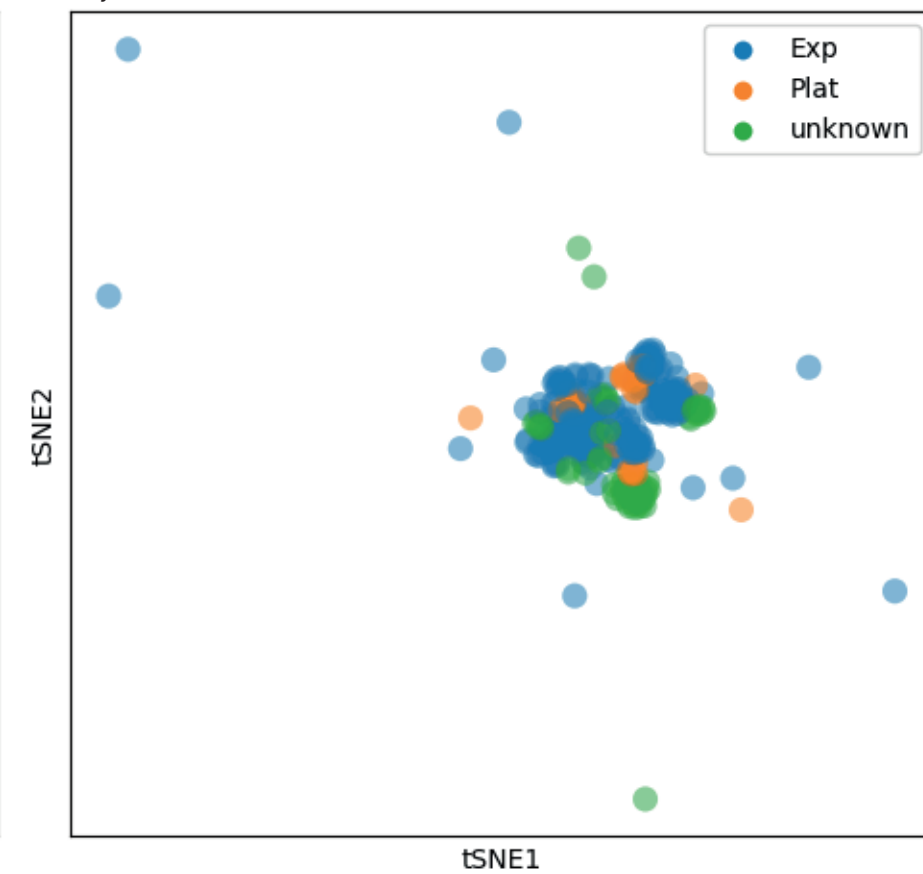

Supplement: vbac071_Supplementary_Data [file vbac071_supplementary_data.zip › vbac071_supplement/supplFigures/normalCombat_RNA.pdf]

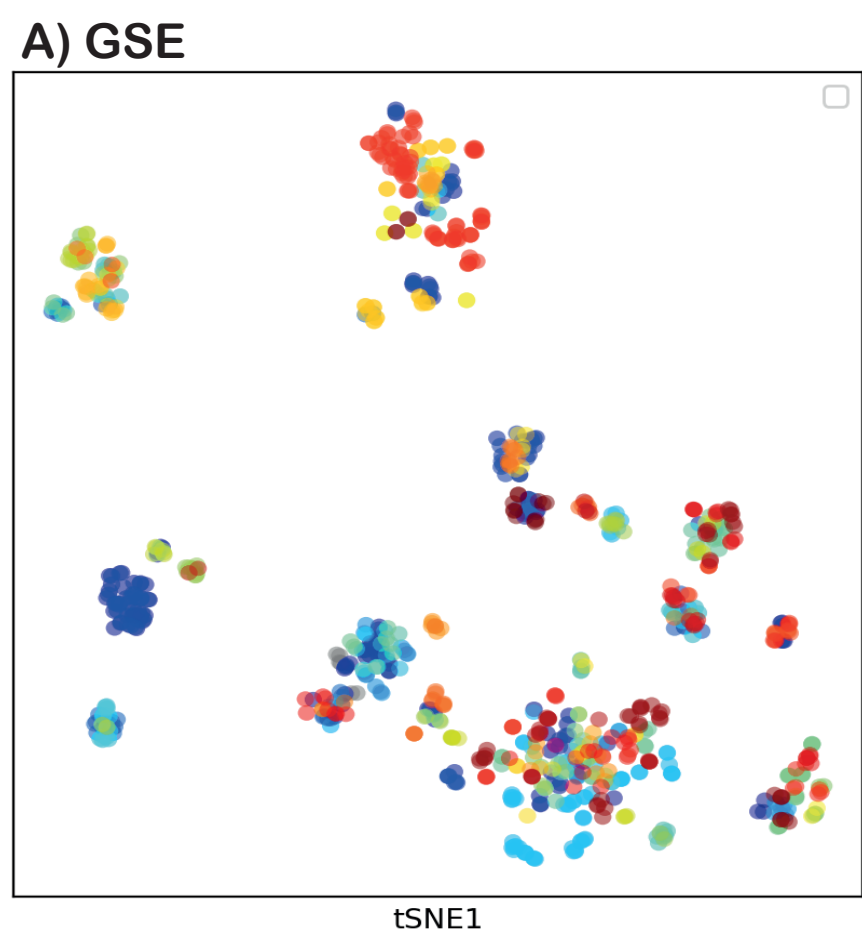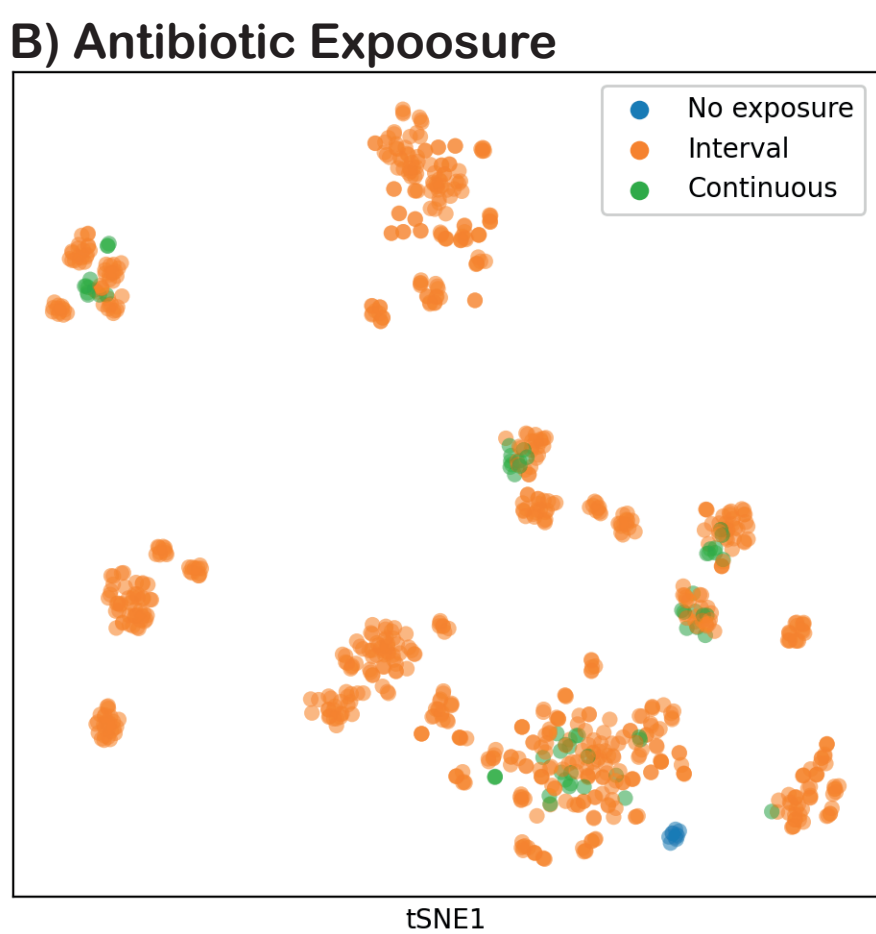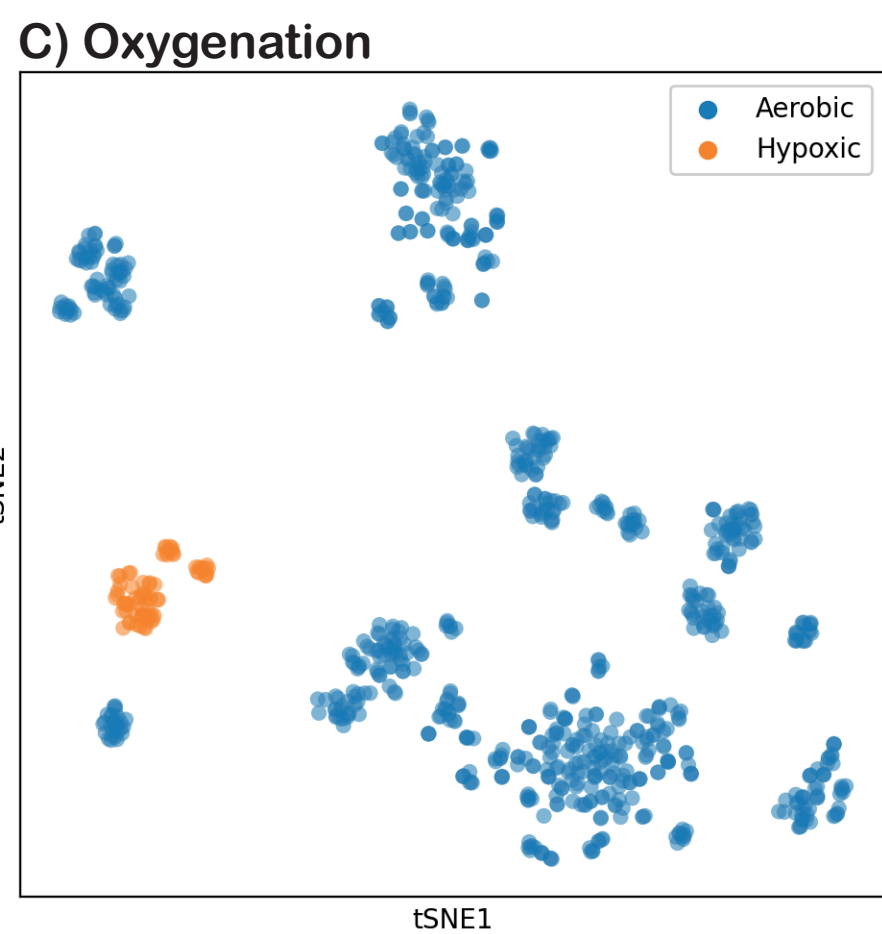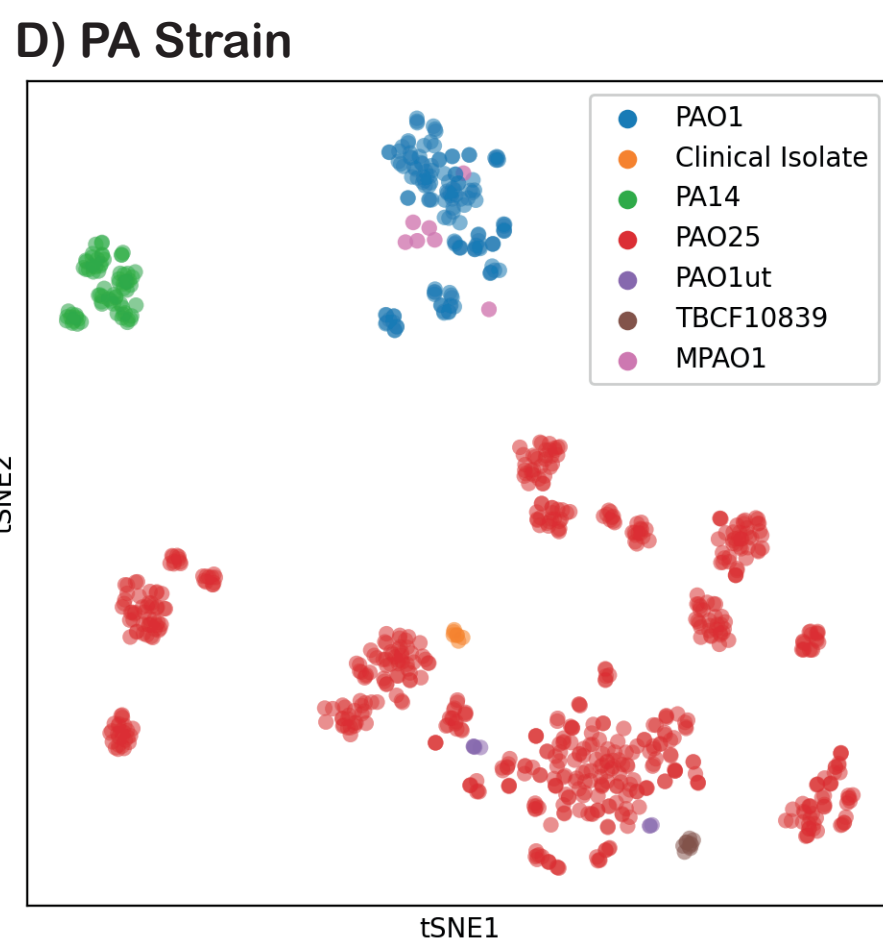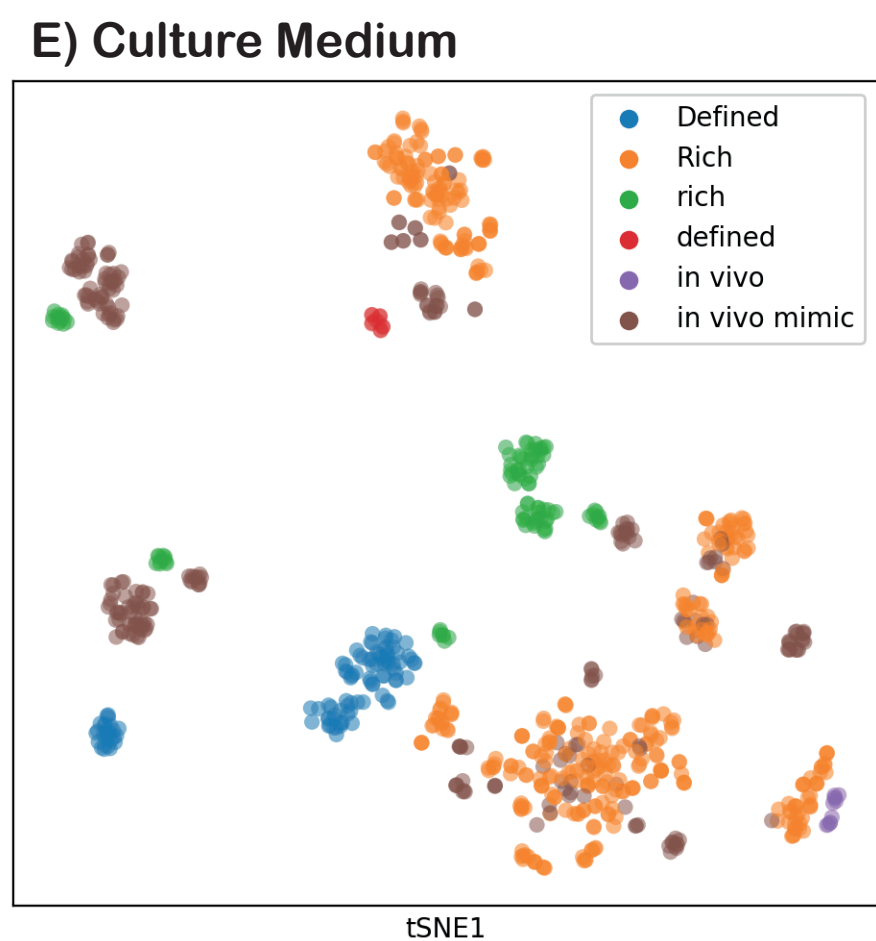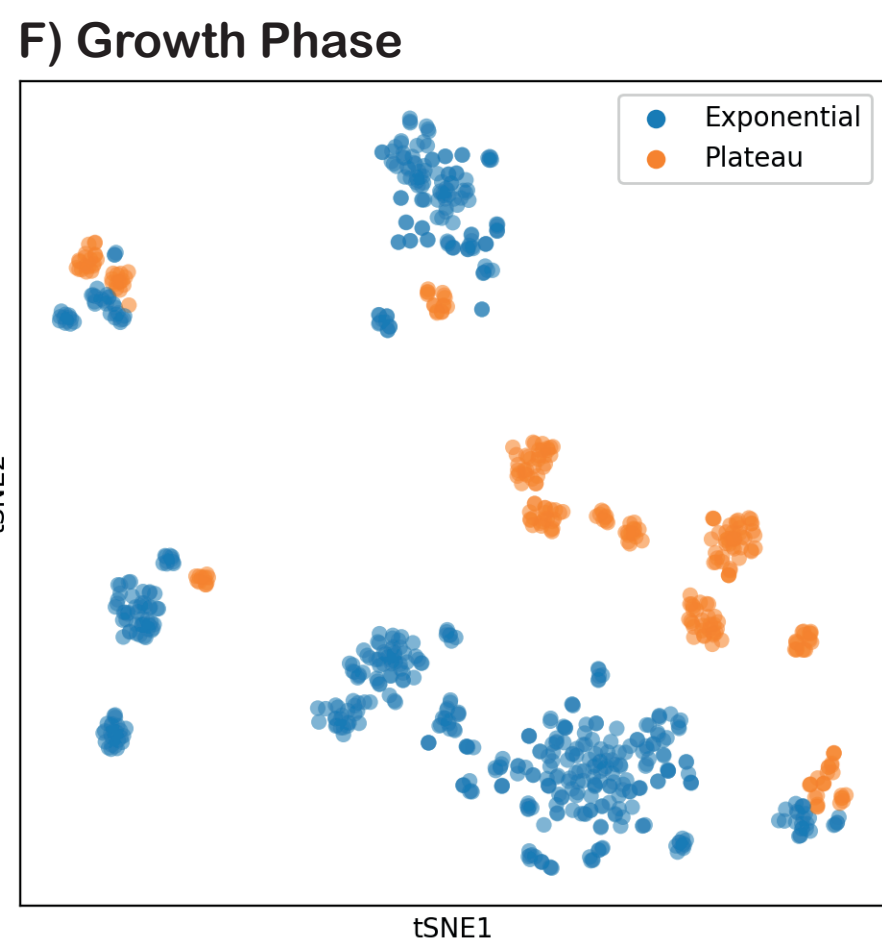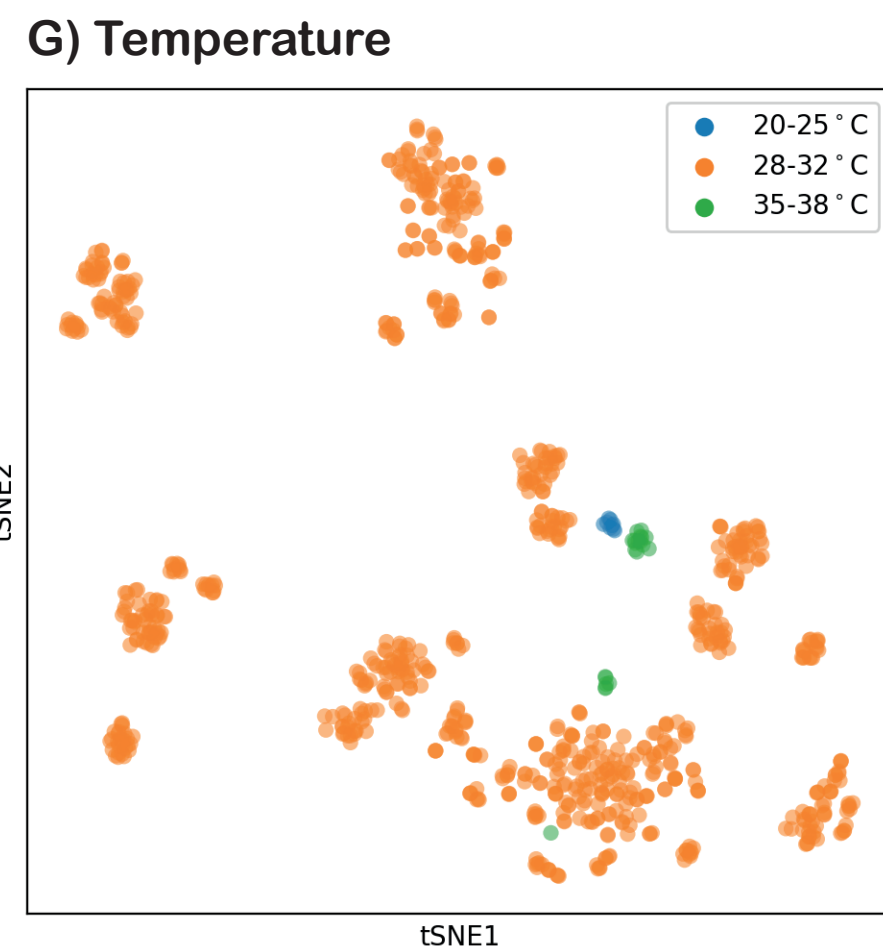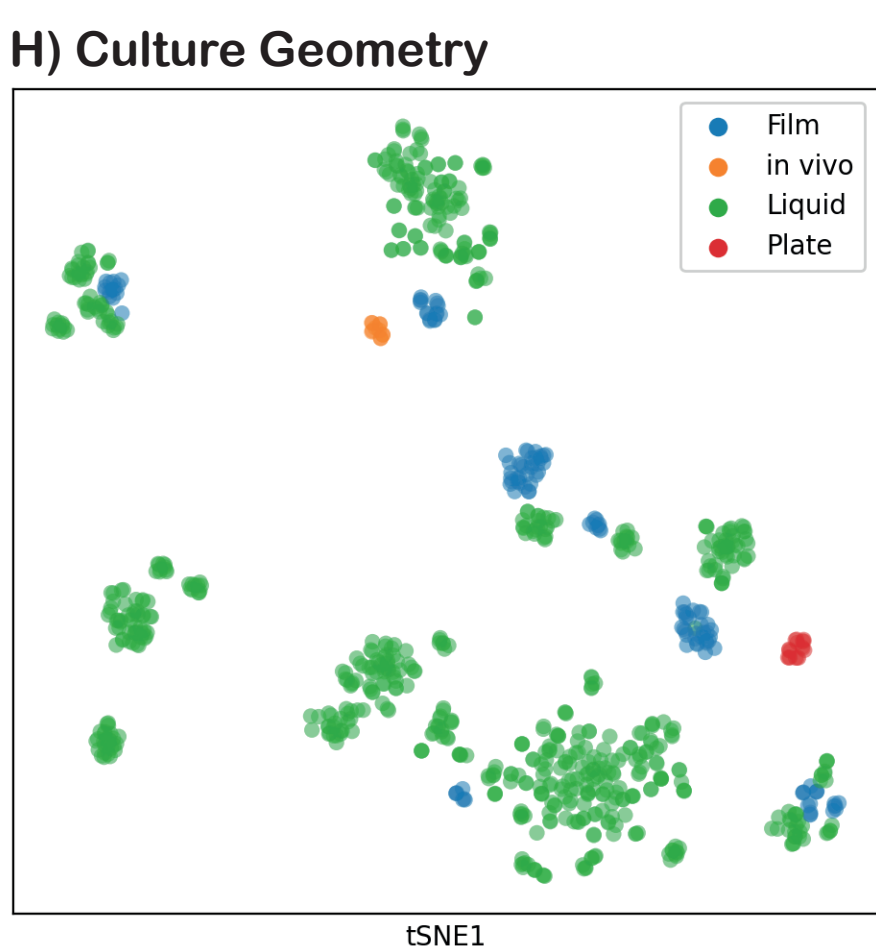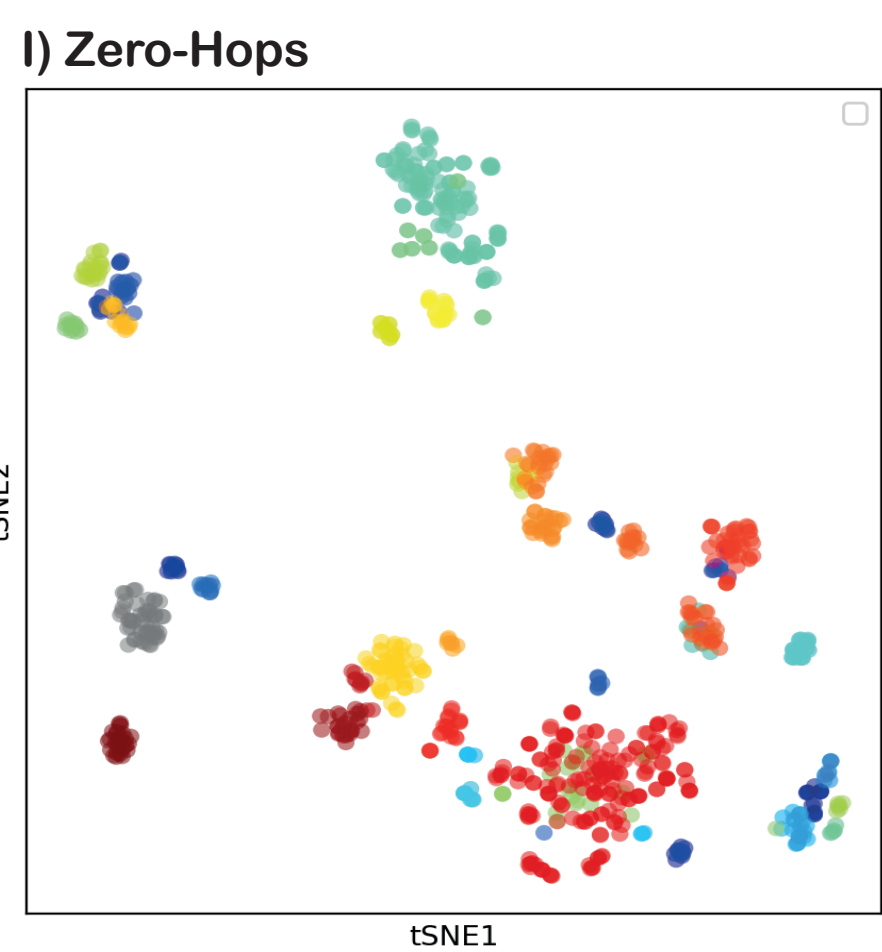

Supplement: vbac071_Supplementary_Data [file vbac071_supplementary_data.zip › vbac071_supplement/supplFigures/PCel.pdf]

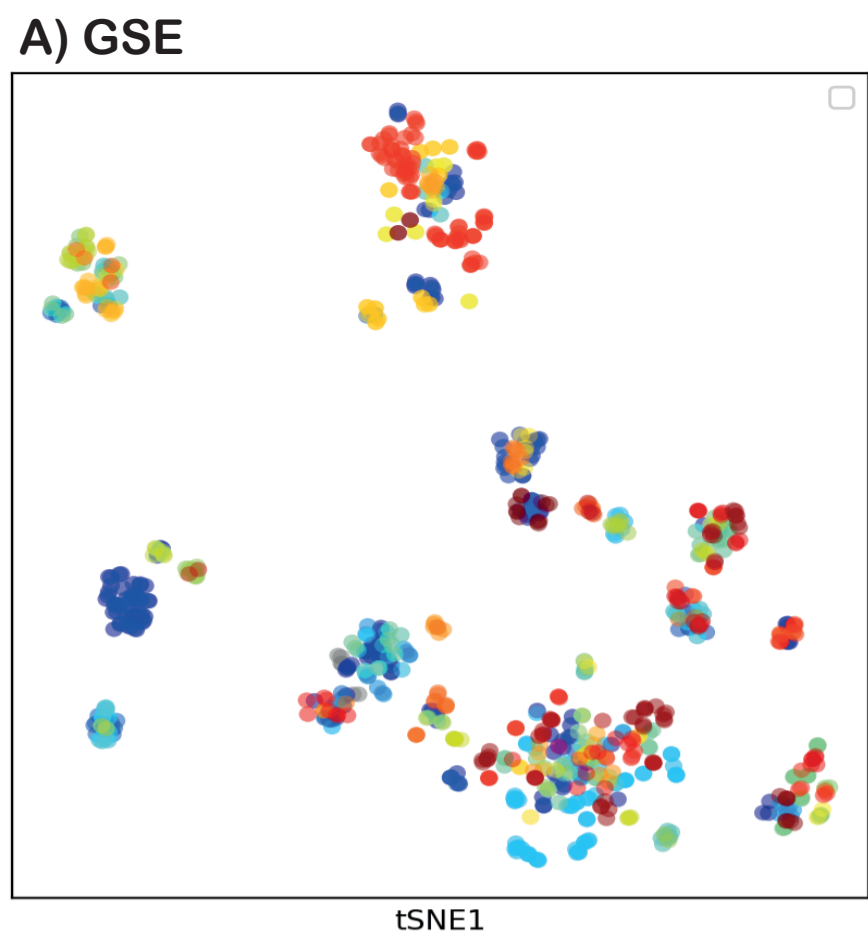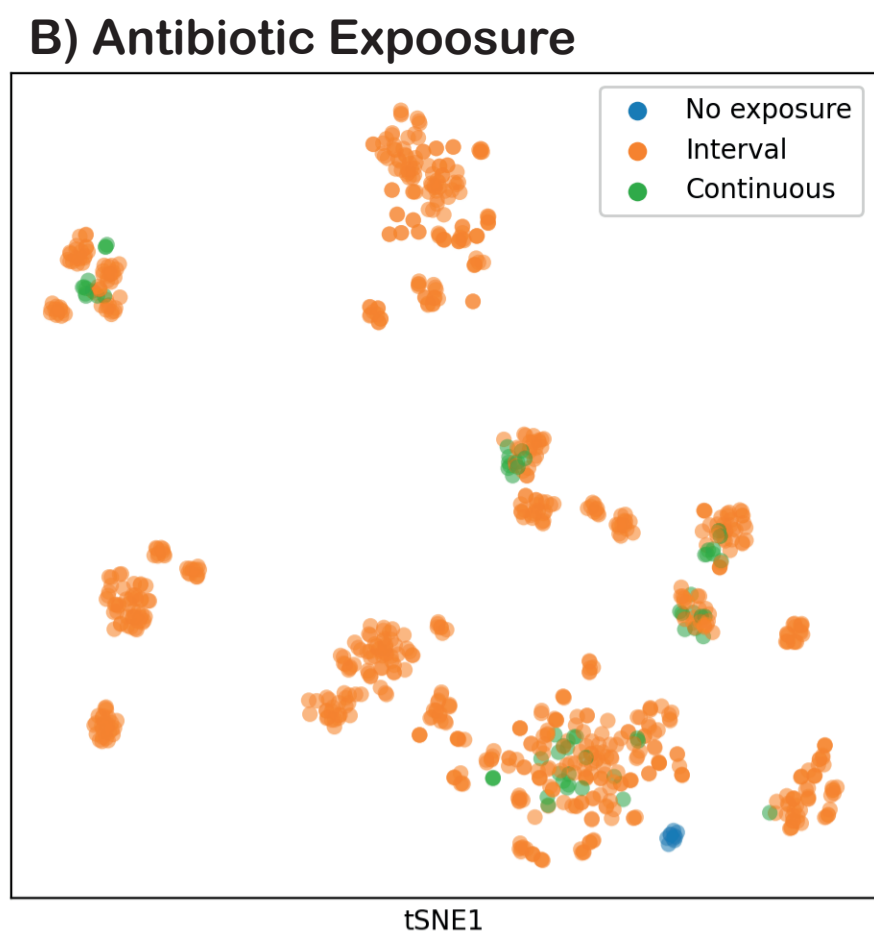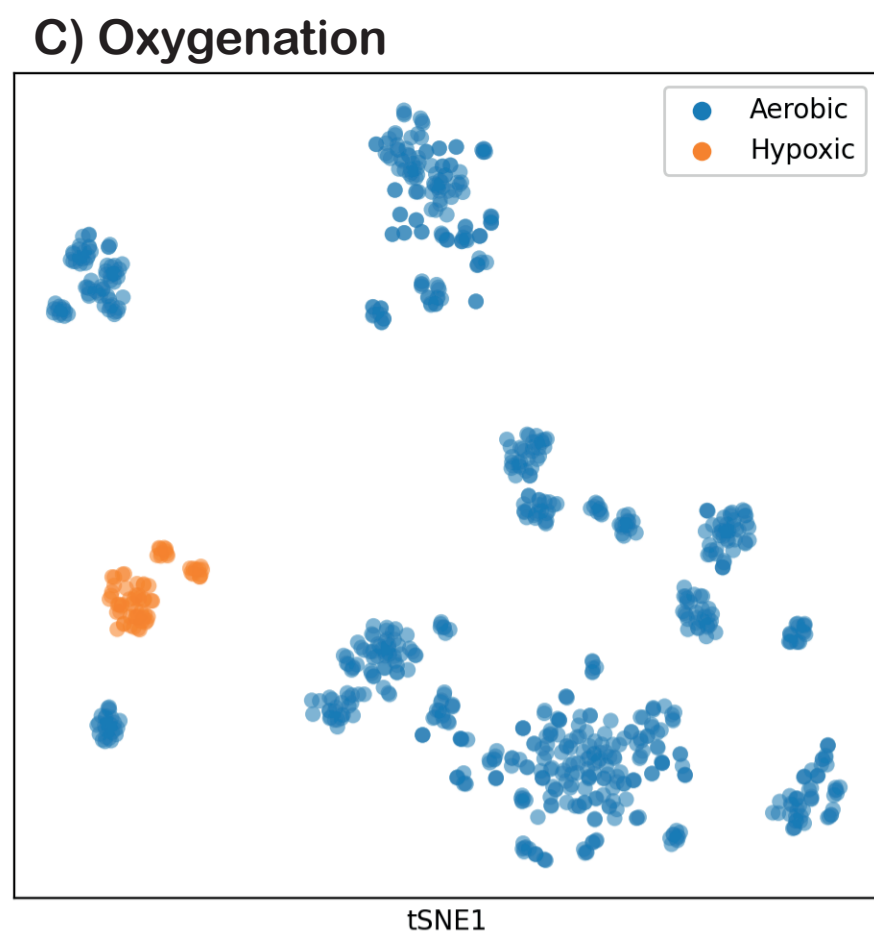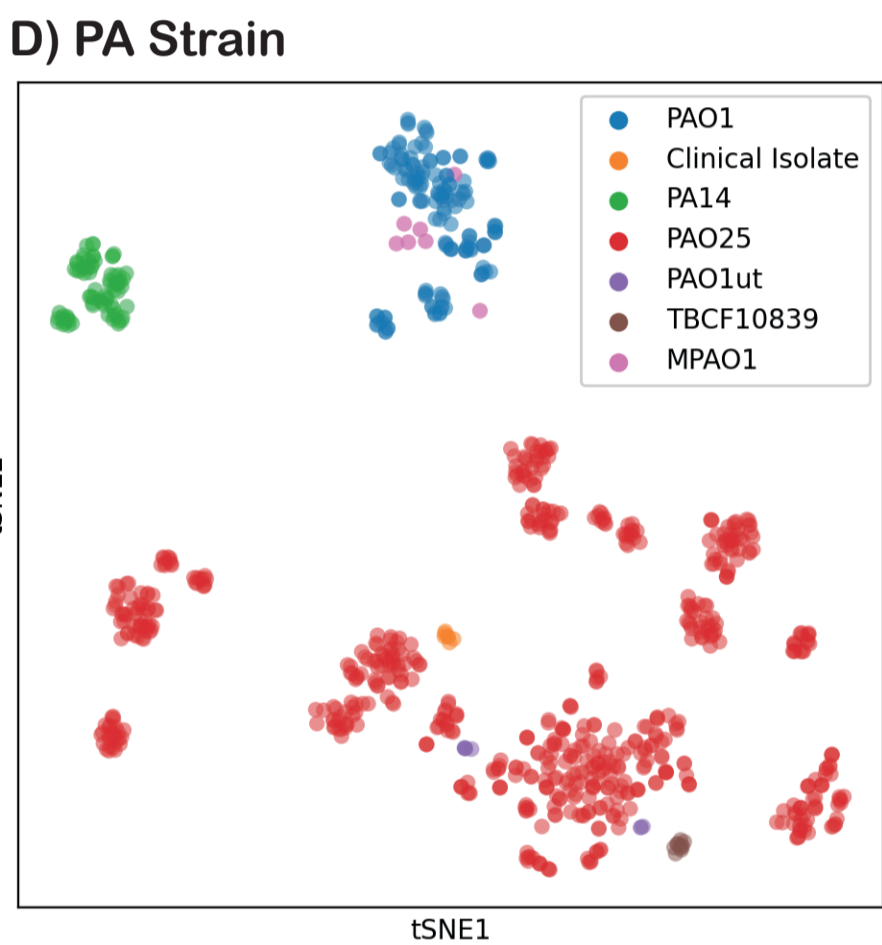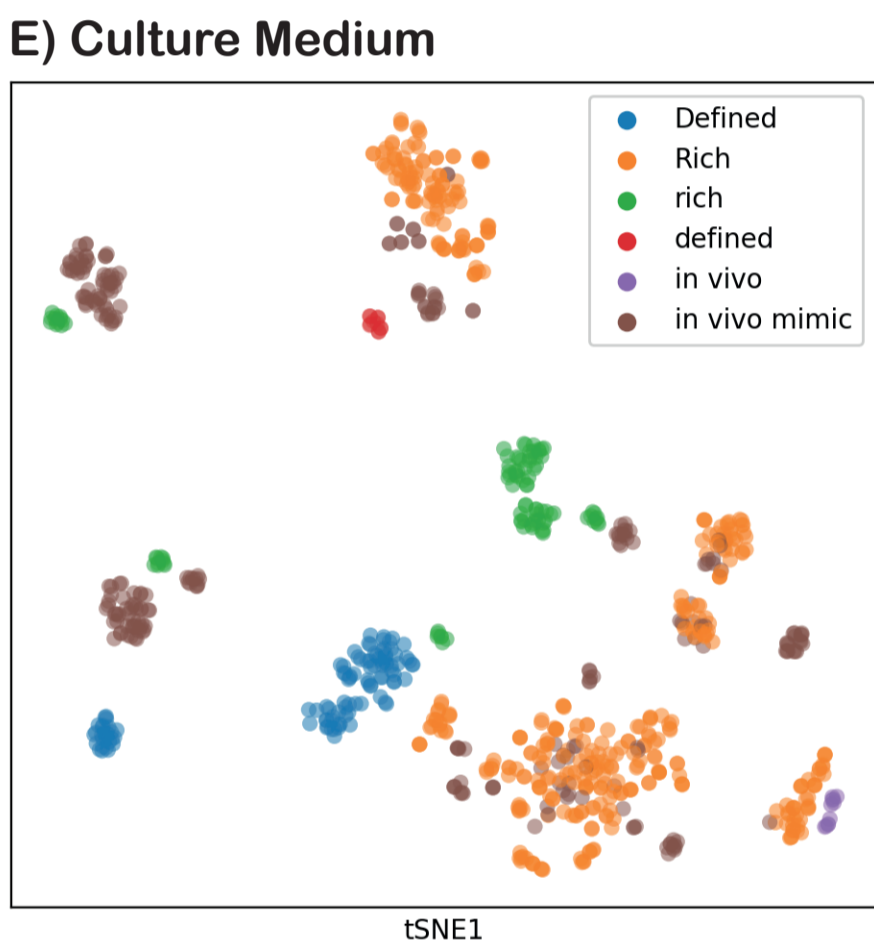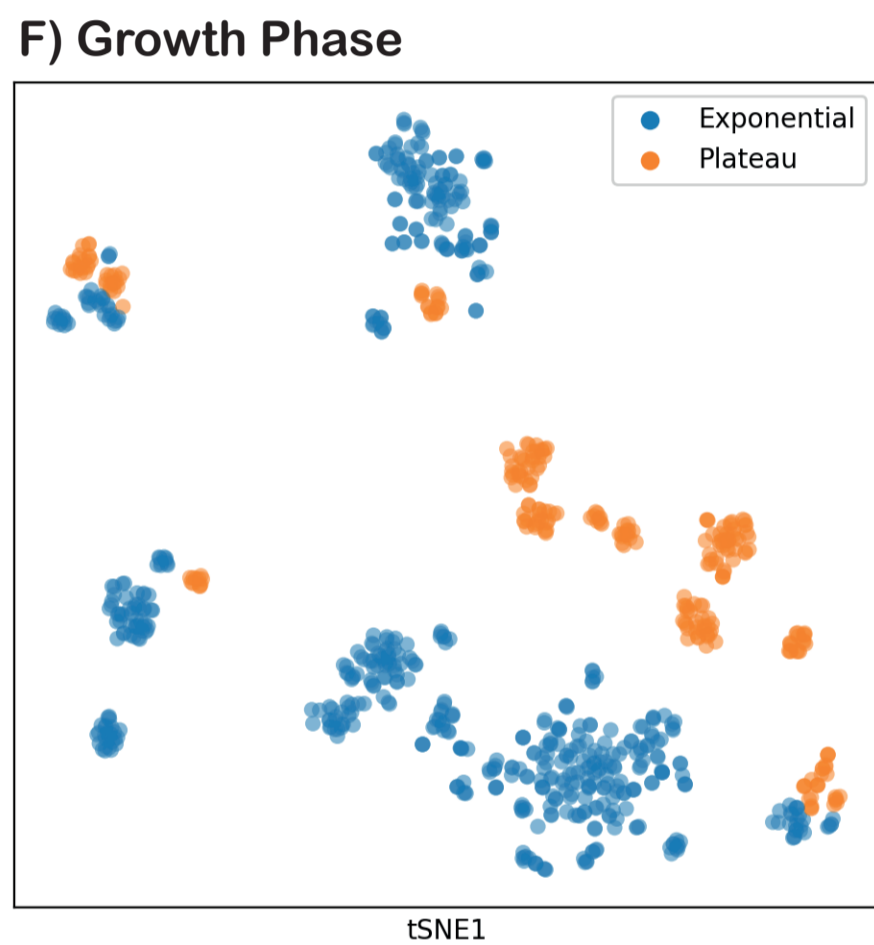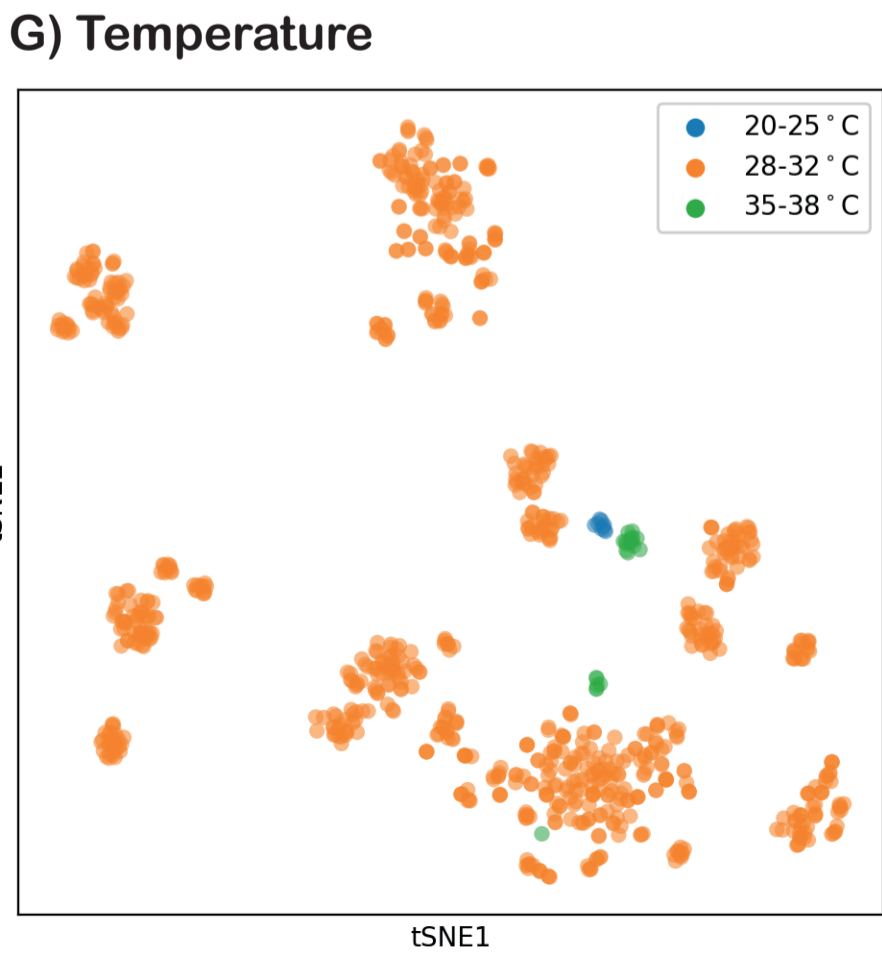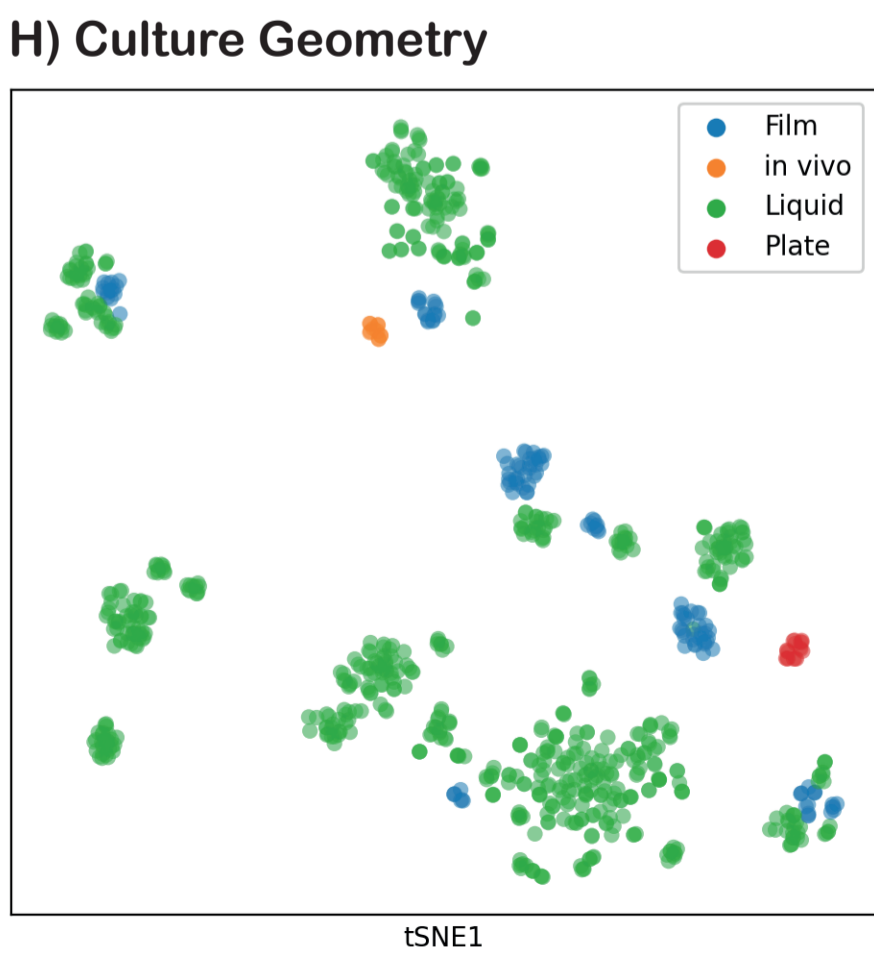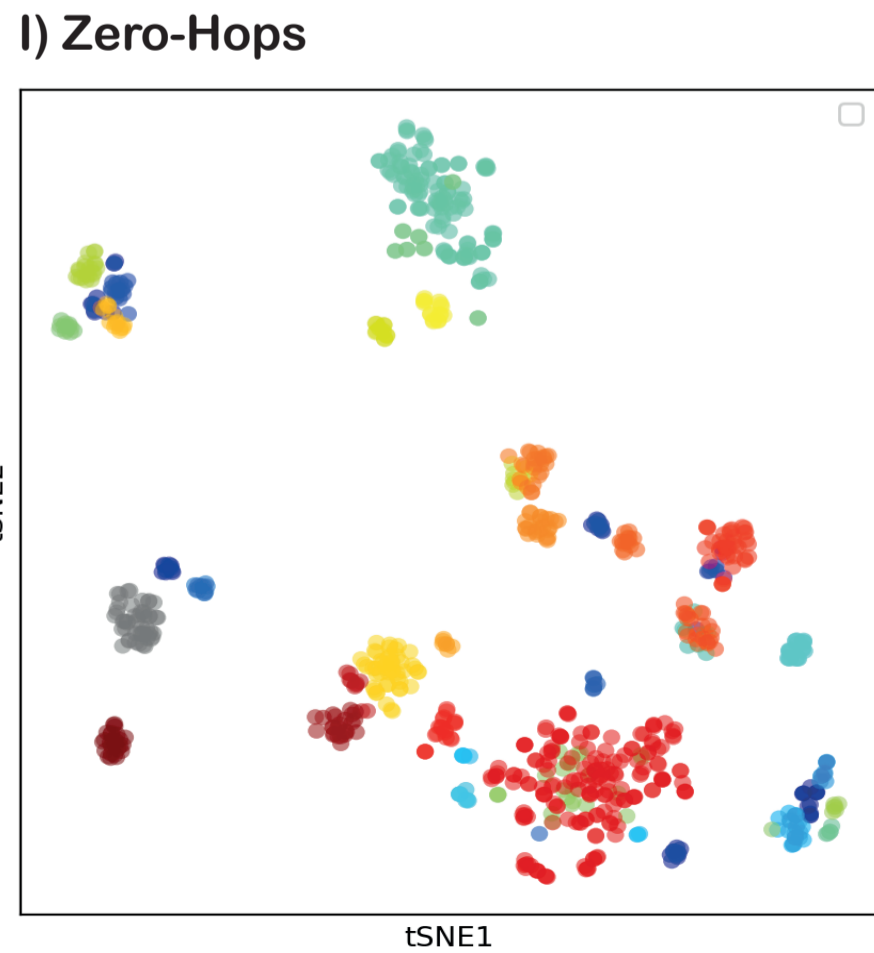

Supplement: vbac071_Supplementary_Data [file vbac071_supplementary_data.zip › vbac071_supplement/supplFigures/reCombat.pdf]
